# Effects of Osteocyte Shape on Fluid Flow and Fluid Shear Stress of the Loaded Bone

**DOI:** 10.1155/2022/3935803

**Published:** 2022-05-30

**Authors:** Fengjian Yang, Weilun Yu, Xuyang Huo, Hongliang Li, Qiuju Qi, Xiaohang Yang, Nianqiu Shi, Xiaogang Wu, Weiyi Chen

**Affiliations:** ^1^College of Biomedical Engineering, Jilin Medical University, Jilin, Jilin, China; ^2^College of Biomedical Engineering, Taiyuan University of Technology, Taiyuan, Shanxi Province, China

## Abstract

This study was conducted to better understand the specific behavior of the intraosseous fluid flow. We calculated the number and distribution of bone canaliculi around the osteocytes based on the varying shapes of osteocytes. We then used these calculated parameters and other bone microstructure data to estimate the anisotropy permeability of the lacunar-canalicular network. Poroelastic finite element models of the osteon were established, and the influence of the osteocyte shape on the fluid flow properties of osteons under an axial displacement load was analyzed. Two types of boundary conditions (BC) that might occur in physiological environments were considered on the cement line of the osteon. BC1 allows free fluid passage from the outer elastic restraint boundary, and BC2 is impermeable and allows no free fluid passage from outer displacement constrained boundary. They both have the same inner boundary conditions that allow fluid to pass through. Changes in the osteocyte shape altered the maximum value of pressure gradient (PG), pore pressure (PP), fluid velocity (FV), and fluid shear stress (FSS) relative to the reference model (spherical osteocytes). The maximum PG, PP, FV, and FSS in BC2 were nearly 100% larger than those in BC1, respectively. It is found that the BC1 was closer to the real physiological environment. The fluid flow along different directions in the elongated osteocyte model was more evident than that in other models, which may have been due to the large difference in permeability along different directions. Changes in osteocyte shape significantly affect the degrees of anisotropy of fluid flow and porous media of the osteon. The model presented in this study can accurately quantify fluid flow in the lacunar-canalicular network.

## 1. Introduction

The cortical bone contains two hierarchical structures of interconnected channels. The larger system comprises Haversian and Volkmann's canals, and the smaller one is the lacunar-canalicular network. Osteocytes are bathed in the interstitial fluid of the lacunar-canalicular porosity, and mechanical loading drives the free fluid in and out of the pore by inducing bone matrix deformation [1]. The pore pressure gradient [2, 3], solute transport [4, 5], and fluid shear stress generated by the fluid flow are considered to be significant biomedical signals for osteocyte mechanotransduction in situ [6–8]. Osteocytes are the most sensitive bone cell type that are considered as mechanosensors within bone that can sense mechanical stimulations and transduce them into biochemical signals [9], thereby regulating bone remodeling [10–12]; however, whether and how these factors generated by the fluid flow as a flow sensor to activate the native osteocyte remains unclear [13]. Strain amplification effect on osteocyte membrane may produce a less osteogenic than response than fluid flow [14], and some studies have suggested that the fluid flow shears the osteocyte membranes or induces cytoskeleton deformation to elicit biochemical responses [1, 6, 8, 15]. In addition, the primary cilium and integrin from osteocytes may be a mechanosensor under the fluid flow [14, 16]. However, osteocytes are embedded in a mineralized extracellular matrix, making it difficult to apply direct experimental approaches. Therefore, mathematical models of fluid flow in the bone matrix have been established [17–20]. Poroelasticity is a well-developed concept for investigating the interaction of fluid and solid phases in the bone [3, 21–23]. Permeability is an important index to describe fluid flow; it determines how fast fluid can flow through the pores. Numerical simulation can be used to explore the fluid flow induced by mechanical loading and calculate the values of pore pressure (PP), fluid velocity (FV), and fluid shear stress (FSS) in the bone using the poroelastic model [3, 4, 7, 8, 23]. Compared with the isotropic model (i.e., osteocytes are spherical), the anisotropic model can more accurately reflect the specific microstructure of bone [24], such as the shape, direction, and density of lacunar-canalicular network [25].

In recent years, the spatial characteristics of osteocytes including morphology and orientation and the potential relationship between these characteristics and disease have gained importance [12, 25–27]. In vitro experiments indicate that the osteocyte geometry affects its strain response [26, 28]. Carter et al. found that the density, shape, and orientation of the osteocytes in the anterior, posterior, medial, and lateral femur significantly differ probably because of local changes in the load [25]. Recent studies have shown that the osteocytes of osteoporosis patients are more irregular and the bone canaliculi are more curved than normal; the FSS and FV on the osteocyte membrane are also greatly altered [29]. Age is an important factor affecting the shape of osteocytes [30]. As age increases, the surface area of osteocytes decreases and the osteocytes flatten [31]. Changes in osteocyte morphology can determine the changes of the three-dimensional distribution of bone canaliculus, resulting in anisotropic permeability of bone tissue [32]. In the finite element analysis of poroelasticity, it is necessary to accurately quantify the permeability of the lacunar-canalicular network to capture the anisotropic fluid flow behavior of the bone.

Therefore, to more accurately elucidate the specific behavior of intraosseous fluid flow, we developed a poroelastic finite element model based on the microstructure of the bone tissue. First, a three-dimensional bone permeability analysis was performed according to the three-dimensional distribution of bone canaliculus calculated from osteocyte shape. Then, based on the theory of poroelasticity, a finite element model of osteons was established to calculate the fluid flow behavior under an axial load. The results are expected to improve our understanding of the mechanism of bone conduction and bone functional adaptation.

## 2. Material and Methods

### 2.1. Calculation of Osteon Permeability Based on Osteocyte Shape

**Figure 1:**
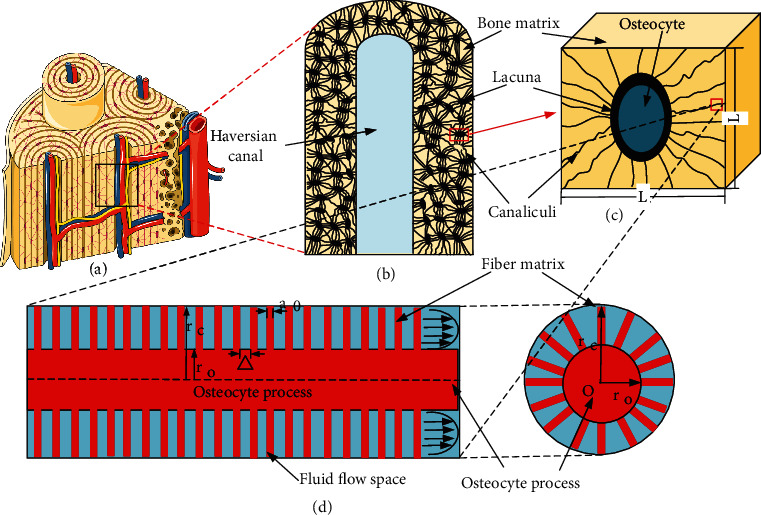
The hierarchical structure of the bone tissue.

The interstitial bone and an osteon cluster are shown in Figure 1(a). A single osteon (among the cluster shown in Figure 1(a)) is shown in Figure 1(b). Assuming a regular arrangement of the lacunar-canalicular network (Figure 1(b)) and uniform distribution of the bone canaliculi, osteons can be considered to compose the cube periodic unit cells (CPUC) that surround the osteocyte lacuna (Figure 1(c)). The microstructure of the canaliculus is shown in Figure 1(d); *r*_c_ is the radius of the canaliculus, *r*_o_ is the radius of the osteocyte process, and *a*_0_ is the radius of the fiber matrix around the osteocyte process.

Expanding the Weinbaum et al. model to account for the 3-D distribution of the canaliculi, the lacuna-canalicular permeability, *k*_lcp_, was calculated based on the anatomical features of the lacuna-canalicular network as follows [7]:

(1) $$\begin{array}{c} k_{\text{lcp}}=\frac{2\pi n_{\text{i}}a^{4}q^{3}}{\gamma^{3}L^{2}}\left\{A_{1}\left[I_{1}\left(\frac{\gamma }{q}\right)-{qI}_{1}\left(\gamma \right)\right]+B_{1}\left[{qK}_{1}\left(\gamma \right)-K_{1}\left(\frac{\gamma }{q}\right)\right]+\frac{\gamma \left(q^{2}-1\right)}{2q}\right\}. \end{array}$$

*q* is the dimensionless ratio between *r*_c_ (0.23 *μ*m) and *r*_o_ (0.1 *μ*m) (*q* = *r*_c_/*r*_o_). *γ* is a dimensionless length ratio between the *r*_c_ and the square root of the small-scale permeability (*k*_p_) constant for the fluid annulus, which is filled with a fiber matrix $\gamma =b/\sqrt{k_{\text{p}}}$ and *k*_p_ = 0.0572 *a*_0_^2^(Δ/*a*_0_)^2.377^, where *a*_0_ is the radius of the pericellular fibers (5 nm) and Δ is the effective spacing of the fibers of the pericellular matrix (7 nm) [7, 18, 32].

*A*_1_ and *B*_1_ can be obtained from the following equation:

(2) $$\begin{array}{c} A_{1}=\frac{K_{0}\left(\gamma \right)-K_{0}\left(\gamma /q\right)}{I_{0}\left(\gamma /q\right)K_{0}\left(\gamma \right)-I_{0}\left(\gamma \right)K_{0}\left(\gamma /q\right)},\\ B_{1}=\frac{I_{0}\left(\gamma \right)-I_{0}\left(\gamma /q\right)}{I_{0}\left(\gamma /q\right)K_{0}\left(\gamma \right)-I_{0}\left(\gamma \right)K_{0}\left(\gamma /q\right)}. \end{array}$$

*I*_*n*_ and *K*_*n*_ denote the first and the second modifications of the Bessel function of order *n*, respectively.

*L* represents the distance between two bone lacunae, which is also the side length of CPUC. It can be obtained from the following formula:

(3) $$\begin{array}{c} L={\left(\frac{V_{L}}{N_{\text{Lac}}}\right)}^{1/3}. \end{array}$$

*V*_*L*_ represents unit volume, and *N*_Lac_ represents the number of lacunae per cubic millimeter of bone unit volume. The range of *N*_Lac_ is 26–90 × 10^3^ (*N*_Lac_/mm^3^) [25, 33]. In this study, the *N*_Lac_ value of unit volume was selected as 37 × 10^3^, so that the value of *L* was 30 *μ*m. According to the literature, the average number of bone canaliculi around each bone lacuna is *N* = 62 [34]. Because the morphology of osteocytes is similar to an ellipsoid, we used the standard equation of an ellipsoid to represent the osteocyte.

(4) $$\begin{array}{c} \frac{x^{2}}{a^{2}}+\frac{y^{2}}{b^{2}}+\frac{z^{2}}{c^{2}}=1. \end{array}$$

The long half axis is *a*, middle half axis is *b*, and short half axis is *c*. The porosity of the lacunar-canalicular network can be expressed by the following formula:

(5) $$\begin{array}{c} \varphi =\frac{\text{N}\pi \left(r_{\text{c}}^{2}-r_{\text{o}}^{2}\right)Lc+4/3\pi abc}{L^{3}}. \end{array}$$

**Figure 2:**
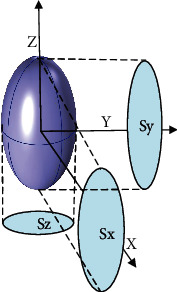
The projection area of osteocytes along the *x* axis (*S*_*x*_), *y* axis (*S*_*y*_), and *z* axis (*S*_*z*_).

*L*_C_ is the average length of the bone canaliculi. We regarded the whole bone lacuna space and osteocyte body as one pore space. Weinbaum et al. assumed that the number of canaliculi passing through the surface of the CPUC in each principal direction was the same [7]. However, because of differences in the osteocyte shape, the number of canaliculi (*n*_*i*_) crossing each face of the CPUC will be anisotropic [32]. As shown in Figure 2, the 3-D distribution of the canaliculi was based on the projection surface area of the osteocyte shape. The number of canaliculi in the different directions could be measured by the projection area ratio of the osteocyte [32]:

(6) $$\begin{array}{c} \left\{\begin{array}{c} n_{x}=\frac{1}{2}\frac{S_{x}}{S_{x}+S_{y}+S_{z}}\times N,\\ n_{y}=\frac{1}{2}\frac{S_{y}}{S_{x}+S_{y}+S_{z}}\times N,\\ n_{z}=\frac{1}{2}\frac{S_{z}}{S_{x}+S_{y}+S_{z}}\times N. \end{array}\right. \end{array}$$

*n*_*x*_, *n*_*y*_, and *n*_*z*_ are the numbers of canaliculi parallel to the *x* (radial), *y* (tangential), and *z* (axial) axes passing through each face of the CUPC, respectively; *S*_*x*_, *S*_*y*_, and *S*_*z*_ are projected surface areas of the bone lacuna in the *x*, *y*, and *z* directions, respectively [32].

### 2.2. Osteocyte Shape

**Figure 3:**
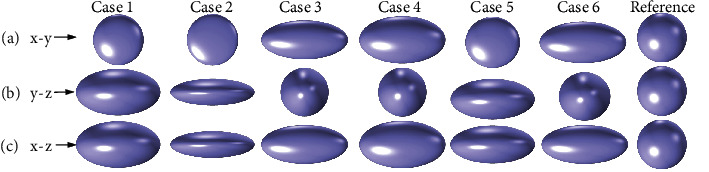
Representative cases of osteocyte shapes. The projected osteocyte shapes are shown schematically in (a) the *x*-*y* plane, (b) the *y*-*z* plane, and (c) the *x*-*z* plane.

To more clearly describe the shape of the osteocyte, we defined three eigenvalues EV1, EV2, and EV3 (EV1 is the square of the long half axis, EV2 is the square of the middle half axis, and EV3 is the square of the short half axis) [25]. The shape parameters were then computed for each ellipsoid based on the resulting three EVs. Three ratios of the EV, degree of anisotropy (1−EV3 : EV1), degree of elongation (1−EV2 : EV1), and degree of flatness (1−EV3 : EV2) were derived from studies of particle shape to define the degree of difference. As shown in Table 1 and Figure 3, several groups of osteocytes with different indicators (anisotropy, elongation, and flatness) were considered to observe the influence of osteocyte shape on the internal fluid flow. Figure 3 shows the importance of these three indicators on the shape of the osteocyte.

When the three eigenvalues were equal (EV1 = EV2 = EV3), the osteocyte was spherical (reference). When EV1 = EV2 > EV3, the osteocyte was flat (Case 1 and Case 2). When EV1 > EV2 = EV3, the osteocyte was elongated (Case 3 and Case 4). When the three eigenvalues were different (EV1 > EV2 > EV3), the shape depended on the extension length and flatness (Case 5 and Case 6).

According to the osteocyte shape parameters (Table 1), the permeability values of Case 1–Case 6 were denoted K1, K2, K3, K4, K5, and K6, respectively. The permeability of the reference model was isotropic, and its value was 1.05 × 10^−20^ m^2^.

(7) $$\begin{array}{c} \text{K}1=\left(\begin{array}{ccc} 8.25\times 10^{-21} & \; & \;\\ \; & 8.25\times 10^{-20} & \;\\ \; & \; & 1.50\times 10^{-20} \end{array}\right)m^{2},\text{K}2=\left(\begin{array}{ccc} 6.10\times 10^{-21} & \; & \;\\ \; & 6.10\times 10^{-21} & \;\\ \; & \; & 1.93\times 10^{-20} \end{array}\right)m^{2},\text{K}3=\left(\begin{array}{ccc} 5.74\times 10^{-21} & \; & \;\\ \; & 1.29\times 10^{-20} & \;\\ \; & \; & 1.29\times 10^{-20} \end{array}\right)m^{2},\\ \text{K}4=\left(\begin{array}{ccc} 6.77\times 10^{-21} & \; & \;\\ \; & 1.28\times 10^{-20} & \;\\ \; & \; & 1.28\times 10^{-20} \end{array}\right)m^{2},\text{K}5=\left(\begin{array}{ccc} 7.31\times 10^{-21} & \; & \;\\ \; & 7.84\times 10^{-21} & \;\\ \; & \; & 1.64\times 10^{-20} \end{array}\right)m^{2},\text{K}6=\left(\begin{array}{ccc} 5.91\times 10^{-21} & \; & \;\\ \; & 1.24\times 10^{-20} & \;\\ \; & \; & 1.32\times 10^{-20} \end{array}\right)m^{2}. \end{array}$$

### 2.3. Establishment of Governing Equation and Finite Element Model of Osteon Governing Equation

**Figure 4:**
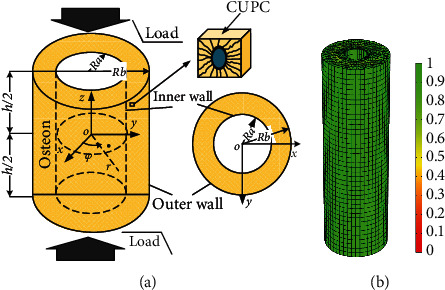
Establishment of the osteon model (a) and mesh generation (b).

Due to the periodicity of the geometrical configuration, we defined the representative elementary volume (REV) by CUPC. The poroelasticity theory efficiently describes the fluid flow behavior of the osteon [3, 4, 7, 19, 35]. The osteon was illustrated as a solid-liquid coupling porous elastic material composed of CUPC units in this study. As shown in Figure 4, the osteon was considered as a hollow annular cylinder that was under cyclic loading in the longitudinal orientation. The following governing equations could describe the poroelastic behavior of the bone, and no body forces were considered. Constitutive laws for the solid matrix material and the saturating fluid were as follows [19, 35]:

(8) $$\begin{array}{c} \sigma =C\varepsilon -\alpha p,\\ p=M\left[\xi -\text{tr}\left(\alpha \varepsilon \right)\right]. \end{array}$$

**σ** is the total stress tensor, **C** is the drained stiffness tensor, **ε** is the total strain tensor, **α** is the Biot effective stress tensor with the same principal orientations as the compliance tensor, *p* is the PP, and *M* is the Biot modulus that links the fluid content variation to the pressure in the absence of solid matrix deformations. *ξ* is the variations in fluid content, and tr() is the trace operator.

The equilibrium equation is given by

(9) $$\begin{array}{c} \rho {\ddot{u}}^{s}-\nabla \cdot \sigma =0. \end{array}$$

*ρ* is the total density, and ${\ddot{u}}^{s}$ is the second derivative of the displacement.The fluid mass conservation equation is given by

(10) $$\begin{array}{c} \frac{\partial \xi }{\partial t}=-\nabla \cdot V. \end{array}$$

Fluid flow was calculated by Darcy's law:

(11) $$\begin{array}{c} V=-k\left(\nabla p+\rho_{f}{\ddot{u}}^{s}\right). \end{array}$$

**V** is the velocity vector, and **k** is the anisotropic permeability tensor, i.e., the textural parameter allowing to quantify the ability of a porous material to transmit fluids through Darcy's law.

After neglecting body forces, the governing poroelastic equations for an anisotropic material in the low-frequency range (such as walking, a few Hertz) were given by plugging (8) into (10) and plugging (9) and (12) into (11):

(12) $$\begin{array}{c} \left.\begin{array}{c} \alpha \nabla p=\nabla \cdot \left(M\varepsilon \right),\\ \frac{1}{M}\frac{\partial }{\partial t}p-\nabla \cdot \left(k\nabla p\right)=-\frac{\partial }{\partial t}\left[\text{tr}\left(\alpha \varepsilon \right)\right]. \end{array}\right\} \end{array}$$

Given the low load frequency, the Haversian canal acts as a reservoir to maintain the normal fluid flow in and out. It was assumed that the vascular pores were no longer saturated, so the pressure on the surface of the Haversian canal was set to 0 (reference pressure). The pore size of the Haversian canal was much larger than that of the bone canaliculus, so the Haversian canal provided space for the fluid pressure to relax when the bone was under mechanical load.

Two boundary conditions representing the physiological environment of the osteon were considered. At the top and bottom of the osteon, the displacement boundary conditions were applied to generate a cyclic compressive load of 1000 *με*, and the radial and tangential displacements were limited on the bottom to prevent rigid displacement.

*BC1*: the cement line of the osteon is not constrained by the interstitial tissue around the osteon, and the liquid can flow in and out freely. The cement line was not impermeable, and it is affected by the time-dependent confining pressure *p*(*t*)(*p*(*b*, *t*)):

(13) $$\begin{array}{c} {\left.\sigma_{rr}^{1}\right|}_{r=a}={\left.p^{1}\right|}_{r=a}=0,\\ {\left.\sigma_{rr}^{1}\right|}_{r=b}={\left.‐p^{1}\left(t\right)\right|}_{r=b},\\ {\left.\partial p^{1}/\partial r\right|}_{r=b}=0. \end{array}$$

*BC2*: the cement line was impermeable, and displacement was constrained:

(14) $$\begin{array}{c} {\left.\sigma_{rr}^{11}\right|}_{r=a}={\left.p^{11}\right|}_{r=a}=0,\\ {\left.u_{r}^{11}\right|}_{r=b}=0,\\ {\left.\partial p^{11}/\partial r\right|}_{r=b}=0. \end{array}$$

The FSS experienced by the osteocyte and its processes was obtained by the following equation [22]:

(15) $$\begin{array}{c} \text{FSS}=\frac{8\mu v_{\text{r}}}{d}. \end{array}$$

*d* is the mean pore diameter:

(16) $$\begin{array}{c} d=\frac{4}{9}\sqrt{\frac{\left(2\text{T}\sum \sum k\left(\;i,j\right)\right)}{\varphi }}. \end{array}$$

*v*_r_ is the interstitial fluid velocity, given by the Dupuit relation:

(17) $$\begin{array}{c} v_{\text{r}}=\frac{Tv}{\varphi }. \end{array}$$

*T* is the tortuosity of the flow path (*t* = 1 for straight channels), and *v* is the value of Darcy velocity [22].

### 2.4. Model Establishment and Calculation

The COMSOL Multiphysics software was used to investigate the poroelastic behavior of the fluid-solid interaction in osteons under an axial compression load. As shown in Figure 4(a), the osteon was defined as a hollow cylinder composed of CPUC, and its material and geometric parameters are shown in Table 2.

The compression loads on the top and bottom of the osteon were both represented by a harmonic displacement (*w*) of amplitude 0.5 *μ*m and a frequency *f*, which resulted in the maximum strain loading *ε* = 0.001 at *t* = 0.5 s, but the maximum pressure and velocity responses were at *t* = 0.25 s [23, 35]:

(18) $$\begin{array}{c} {\left.w\right|}_{z=\pm 0.5\text{mm}}=\pm 0.00025\left[\cos\left(2\pi ft\right)-1\right]\left[\text{mm}\right]. \end{array}$$

As shown in Figure 4(b), the mesh used in the finite element simulation contained 20880 elements and 54277 degrees of freedom.

To simulate oxygen consumption by the osteocytes, a reaction of osteocyte respiration was incorporated. The convective-diffusive-reactive in lacuno-canalicular was incorporated by [22]

(19) $$\begin{array}{c} \frac{d}{dt}\left(\varphi \rho C_{i}\right)=\nabla \cdot J_{i}+S+R, \end{array}$$

(20) $$\begin{array}{c} J_{i}=\nabla \cdot \left(D_{\text{C}}+D_{\text{d}}\right), \end{array}$$

(21) $$\begin{array}{c} D_{\text{C}}=-\frac{\rho Ck}{u}\nabla p, \end{array}$$

(22) $$\begin{array}{c} D_{\text{d}}=-\phi D\nabla \left(\rho {\text{C}}_{i}\right), \end{array}$$

where *C*_*i*_ is the concentration of the reactants, *S* is some reaction product, *D*_C_ is the convective flux, *D*_d_ is diffusive flux, and *D* is the species' diffusion coefficient. *R* is the reaction of the oxygen consumption, and the released energy exists in *S*:

(23) $$\begin{array}{c} C_{6}{\text{H}}_{12}{\text{O}}_{6}+6{\text{H}}_{2}0+6{\text{O}}_{2}\overset{\text{e}}{⟶}6{\text{CO}}_{2}+12{\text{H}}_{2}\text{O}+\text{energy}. \end{array}$$

Whether glucose or oxygen is transported from the Haversian canal and the Folkman's canal into the lacuno-canalicular network and then consumed by osteocytes remains unclear. In addition, because of consumption, the concentration of glucose and oxygen in the blood is higher than that in the lacuno-canalicular network. The concentration of glucose flowing from Haversian canal into the lacuno-canalicular network is 7.5 mol/m^3^, and the diffusion coefficient is 1 × 10^−10^ m^2^/s [22]. It is worth noting that the oxygen enters into the lungs through respiration, and some combines with hemoglobin (Hb) to form the oxyhemoglobin (HbO_2_), and some dissolve in the blood (PaO_2_). The concentration of oxygen in the blood is about 7.5 mol/m^3^, and the diffusion coefficient is 2.57 × 10^−9^ m^2^/s. The consumption rate of glucose is 1 × 10^−16^ mol/s [22], and the consumption rate of oxygen is 6 × 10^−16^ mol/s. The glucose and oxygen were transported into the lacuno-canalicular network from the Haversian canal and the Folkman canal and outflow from the cement line. The potential for the osteocytes to consume the oxygen was quantified by the amount of carbon dioxide production by the osteocytes (i.e., the concentration of the reaction product in the lacunae).

## 3. Results

This study analyzed the differences in PG, PP, FV, and FSS caused by the changes of osteocyte shape, which are examined in sequence here.

### 3.1. Fluid Pressure Gradient (PG)

**Figure 5:**
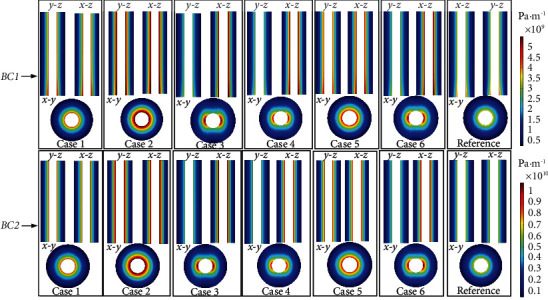
The magnitude of pressure gradient distribution under different boundary conditions at *t* = 0.25 s. BC1: elasticity restrained. BC2: displacement constrained.

**Figure 6:**
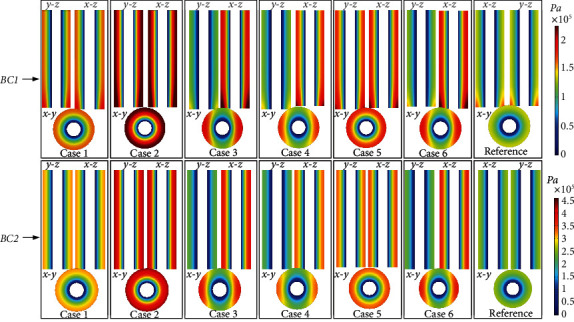
The magnitude of pore pressure distribution under different boundary conditions at *t* = 0.25 s. BC1: elasticity restrained. BC2: displacement constrained.

**Figure 7:**
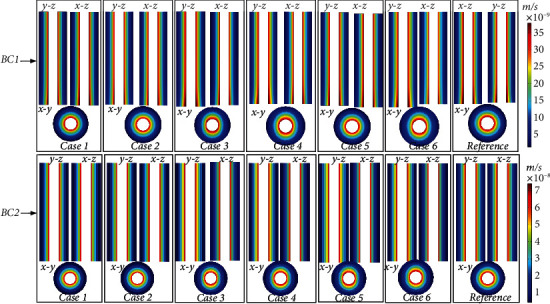
The magnitude of flow velocity distribution under different boundary conditions at *t* = 0.25 s. BC1: elasticity restrained. BC2: displacement constrained.

As shown in Figure 5, the distribution of PG magnitude in the osteon under different boundary conditions at *t* = 0.25 s was plotted. PG refers to the change in pressure per unit length along the direction of fluid flow. It is one of the main driving forces of fluid flow and other effects (FSS, streaming potential, and solute transport) in the osteon [3, 4, 35, 36]. Under the axial symmetrical load, the distribution of the PG magnitude in Case 1–Case 6 was different from that in the reference model, but the values were in same order of magnitude. The maximum PG in Case 1–Case 6 was 5.49*e*9 Pa/m in BC1, whereas it was 3.3*e*9 Pa/m in the reference model. The maximum PG in Case 1–Case 6 was 61.95% larger than that in the reference model. The maximum PG in Case 1–Case 6 in BC2 was 1.08*e*10 Pa/m, whereas it was 6.53*e*9 Pa/m in the reference model. The maximum PG in Case 1–Case 6 was 65.39% larger than that in the reference model. This showed that the osteocyte shape affects the distribution of PG magnitude under the same osteocyte volume, indicating that when the volume of the osteocyte was the same, circular osteocytes exhibited smaller PP and FV. As shown in Figures 6 and 7, the distribution of PP and FV along the *y*-*z* and *x*-*z* planes in Case 1, Case 2, and Case 5 was similar, and this distribution was similar to that in the reference model. Therefore, the three-dimensional distribution of bone canaliculus of elongated osteocytes was more likely to cause the anisotropy of fluid flow in the bone. The maximum value of PG in BC2 was about twice that in BC1. This indicated that the right choice of boundary conditions is essential for understanding fluid flow in the bone.

### 3.2. Pore Pressure and Fluid Velocity

Figures 6 and 7, respectively, show the distribution of PP and FV under different boundary conditions. Due to the different shapes of the osteocyte, the distribution of the PP and FV in Case 1–Case 6 was significantly different from that in the reference model.  Figure 6 shows that the maximum PP value (2.23*e*5 Pa) in Case 1–Case 6 in BC1 was 67.67% larger than that in the reference model (1.33*e*5 Pa). The maximum PP value (4.36*e*5 Pa) of Case 1–Case 6 in BC2 was 67.67% larger than that in the reference model (2.58*e*5 Pa). The maximum PP in BC2 was 95.51% higher than that in BC1.  Figure 7 shows that the maximum FV value (3.76*E*-8 m/s) in Case 1–Case 6 in BC1 was 8.6% larger than that in the reference model (3.46*E*-8 m/s). The maximum FV value (7.44*E*-8 m/s) in Case 1–Case 6 in BC2 was 8.4% larger than that in the reference model (6.86*E*-8 m/s). The maximum FV value in BC2 was 97.87% larger than that in BC1.

### 3.3. Fluid Shear Stress

**Figure 8:**
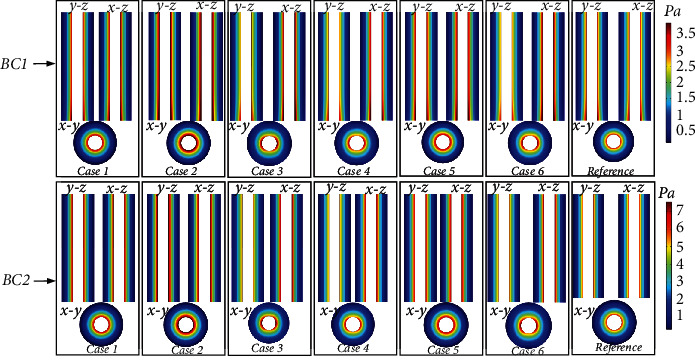
The magnitude of fluid shear stress distribution under different boundary conditions at *t* = 0.25 s. BC1: elastic restrained. BC2: displacement constrained.

Figure 8 shows the distribution of FSS in the osteon with different osteocyte shapes at *t* = 0.25 s. Figures 7 and 8 show similar trends in spatial distribution. In BC1, the maximum FSS value in Case 1–Case 6 was 3.83 Pa, whereas it was 3.0 Pa in the reference model. In BC2, the maximum FSS value in Case 1–Case 6 was 7.55 Pa, whereas it was 5.85 Pa in the reference model. Thus, a change in osteocyte shape would make the maximum FSS value 26.6% and 29% larger than that in the reference model in BC1 and BC2, respectively.

**Figure 9:**
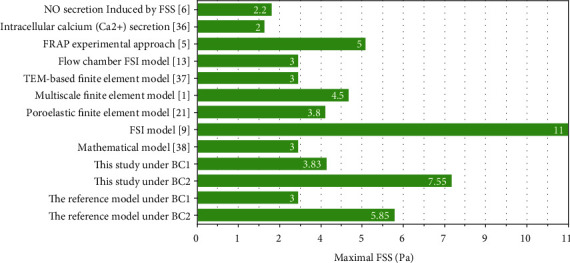
Comparison of FSS measured with different methods.

In order to verify the correctness of the model, we compare the FSS with other simulated or experimental results (Figure 9). In in vitro experiment, the generation of nitric oxide (NO) [6], prostaglandin (PGE2), and osteopontin is in a range of 0.1–2.2 Pa of FSS [6], and the FSS threshold intracellular calcium (Ca^2+^) production was 2 Pa [37]. However, the osteocytes experience FSS on its surface up to 3 Pa. The maximum FSS (3 Pa) of our reference model under BC1 is consistent with the results of poroelastic finite element model and FSI model [14, 38, 39]. The FSS under BC2 obtained in this study is about 5.85-7.55 Pa, which was comparable to the experimental value of experimental approach based on fluorescence recovery after photobleaching (FRAP) and multiscale finite element [1, 5].

### 3.4. Oxygen Consumption

**Figure 10:**
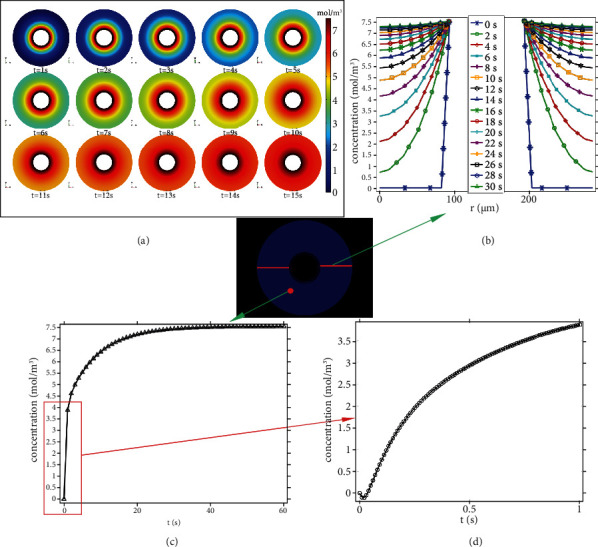
Oxygen concentration distribution. (a) Distribution of oxygen concentration in the osteon cross section at different times (at 1 s-15 s). (b) Concentration profiles describing the oxygen concentration across the osteon cross section. (c) Concentrations of oxygen of one point on osteon as functions of time (s). (d) Local amplification of (c).

**Figure 11:**
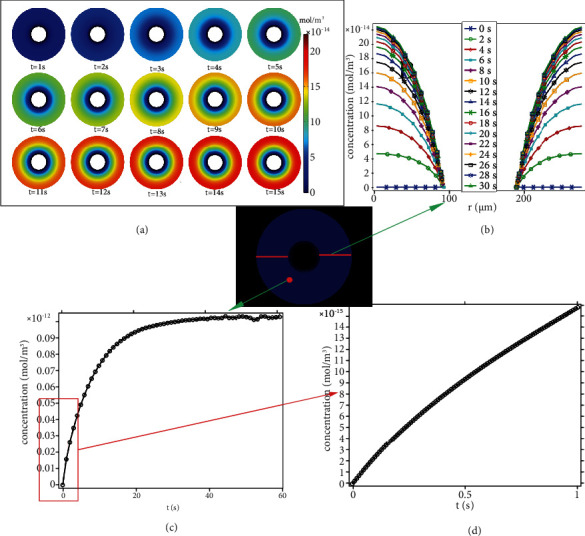
Oxygen consumption distribution. (a) Distribution of oxygen consumption concentration in the osteon cross section at different times (at 1 s-15 s). (b) Concentration profiles describing the oxygen consumption across the osteon cross section. (c) Concentrations of oxygen consumption of one point on osteon as functions of time (s). (d) Local amplification of (c).

Solving the space-dependent mass balances of Equation (19) results in concentration distributions of oxygen concentration and carbon dioxide production as functions of time. Figures 10 and 11 show the space-time-dependent concentration transients of the oxygen concentration and oxygen consumption in an osteon, respectively.

## 4. Discussion

In this study, poroelastic finite element models were developed to investigate the effect of osteocyte shape on fluid flow and FSS in osteons under different boundary conditions. These models were established based on the osteon microstructure to simulate interstitial fluid flow arising from the mechanical deformation of the osteon and PGs under axial loading representative of physical activity. For modeling purposes, we assumed that the osteons were composed of CPUC, and then, we estimated the permeability and porosity of the osteon by estimating the number and three-dimensional distribution of bone canaliculi in different shapes of the bone lacuna.

PG refers to the change in pressure per unit length along the direction of fluid flow. Previous studies have often not discussed this vital parameter [3, 19, 23, 35]. Mechanical loading in the osteon occurs at the whole-organ level, with compression and tension occurring in different regions, driving fluid flow in the lacunar-canalicular network [40]. Maximal PP occurs at the cement line. Because of low blood pressure in Haversian canal boundary, the PP magnitudes maintained to be at a lower level. Therefore, a PG established across the osteon wall should be large enough to drive fluid against the transcortical pressure difference [22]. In this study, the transcortical pressure difference was at least 1.33*e*5 Pa and 2.58*e*5 Pa in the reference model in BC1 and BC2, respectively, and the PG was sufficient (at least 3.3*e*9 Pa/m and 6.53*e*9 Pa/m) to counter the transcortical pressure difference. As shown in Figure 5, the PG decreased dramatically away from the Haversian canal. As a result, osteocytes far away from the Haversian canal had significantly lower FSS than osteocytes relatively close to the Haversian canal (Figure 8).

PP is an essential load-inducing phenomenon in the lacunar-canalicular network, which affects the growth, differentiation, and material transport of osteocytes [3, 4, 22, 41]. The PP changed significantly with osteocyte shape. Specifically, the distribution of PP between Case 3, Case 4, and Case 6 in the *x* and *y* directions was markedly asymmetric, whereas it was axisymmetric in Case 1, Case 2, Case 5, and the reference model in *x* and *y* directions in both BC1 and BC2. This shows the anisotropy of permeability induced by the change in osteocyte shape. The permeability of Case 3, Case 4, and Case 6 models was one order of magnitude different in the *x* and *y* directions, whereas the permeability of Case 1 Case 2, Case 5, and the reference model in the *x* and *y* directions showed little difference. In the *z* direction, the PP of all models did not change substantially. This is because the mechanism of load-induced PP makes the fluid flow into the Haversian canal through the lacunar-canalicular network and release the PP [22]. Therefore, the main fluid flow of osteon is between the cement line and the Haversian canal, and there is almost no fluid flow in the *z* direction. As shown in Figures 5–8, different boundary conditions have significant effects on the flow behavior in the osteon. The maximum PG, PP, FV, and FSS in BC2 were 96.72%, 95.51%, 97.87%, and 97.13% larger than those in BC1, respectively.

In BC1, some physiological pressure generated outside the osteon can neutralize the PP of the osteon, and the outer wall of the osteon is not constrained by the interstitial tissue around the osteon. Therefore, the outer wall of the osteon is only affected by the fluid pressure in the interstitial tissue. In BC2, the cement line of the osteon is constrained by the interstitial tissue around the osteon and cannot move, and no fluid will be allowed across the outer restraint boundary. Some studies have observed that there are bone canaliculi passing through the cement line [20, 42], which indicates that the cement line is indeed permeable and that fluid exchange between the osteon and the external interstitial bone is possible. Therefore, BC1 seems to more closely mimic the physiological state than BC2.

Verbruggen et al. observed the mean interstitial FV (~60.5 *μ*m/s) and the mean maximum FSS (~11 Pa) around osteocytes in vivo by applying a load (3000 *με* compression and 300 Pa PG) representing strong physiological activity [9]. Our result for BC2 was similar to their result; however, the loading in BC2 represents normal physiological activities. Some studies considered that the FSS level required for bone growth is 0.8 Pa [9, 43]. FSS in the range of 0.1–2.2 Pa can increase the production of nitric oxide, prostaglandin, and osteopontin [6, 9]. An FSS of 2 Pa can increase intracellular calcium (Ca^2+^), and an FSS of 0.2–6 Pa can induce cell response [8, 9]. Our results (~3.83 Pa in BC1 and~7.55 Pa in BC2) suggest that the fluid slow stimulating the osteocytes was sufficient to elicit biochemical signals for bone formation. Similar FSS values (~5 Pa) have also been suggested by tracer studies [5, 9]. Our findings reveal that osteocyte shape significantly influences the osteocyte fluid flow.

At a loading frequency (such as walking) of 1 Hz, the load-induced fluid flow should be considered as fluid oscillating back and forth. There was an inflow of simulated glucose, oxygen, and water from the Haversian canal and Volkmann canal, the amount of oxygen consumed by the osteocytes—quantified by the amount of carbon dioxide product in osteon cross section (Figure 11). As shown in Figures 10 and 11, an obvious transosteonal gradient in oxygen concentration and carbon dioxide generation was found before 15 loading cycles (*t* = 15 s). After the 15-cycle loading regime, the variations of oxygen concentration and carbon dioxide generation were beginning to stabilize. The oxygen concentration was almost linearly decreased, and the oxygen consumption was almost linearly increased near the Haversian canal. However, the rate of oxygen concentration decreases and the rate of oxygen consumption near the cement line was significantly reduced. The distance of fluid transport might be the reason that causes the decreased efficiency of transport near the cement line. Generally, many drug delivery systems have been developed through blood flow in vivo. However, the ability to predict and control the rate of release from delivery systems is still a challenge. In targeted drug delivery involving the lacuno-canalicular system, the effects of hemodynamic need to be considered [44, 45].

One limitation of our research is that the canaliculus was idealized as a straight tube. This study does not consider the effect of the curvature of the canaliculus, while in fact, the processes of osteocytes extend through the curved canaliculus from the osteocyte body to the surface of CUPC [9, 36]. Another limitation is that the osteon was considered to be composed of identical CUPC. The shape of the osteocyte in each CUPC may be different, which will lead to a change in the local fluid flow. Theoretically, it is necessary to determine the shape of osteocytes in each CUPC; however, it is observed in the experiment that the shape of bone lacuna is similar in a certain region of bone tissue, and such a region is large enough to contain one or several osteons [25]. Therefore, as long as the osteocyte shape in a specific region of bone is determined, the method of this study can be applied to analyze the load-induced FSS and other fluid flow behaviors.

## 5. Conclusion

In this study, a method was proposed to estimate the anisotropic permeability of the lacunar-canalicular network based on the shape of osteocytes. The fluid flow in the osteon was described under different boundary conditions according to the calculated permeability. The findings can be summarized as follows: (1) changes in osteocyte shape (Case 1–Case 6) make the maximum value of PG, PP, FV, and FSS 33.36%, 67.67%, 8.6%, and 26.6% larger than those in the reference model in BC1 and 65.39%, 67.67%, 8.4%, and 29% larger than those in the reference model in BC2, respectively. (2) The maximum PG, PP, FV, and FSS in BC2 were 96.72%, 95.51%, 97.87%, and 97.13% larger than those in BC1, respectively. (3) The permeability of Case 3, Case 4, and Case 6 had a difference of one magnitude order in the *x* and *y* directions, indicating that elongated osteocytes are more likely to cause anisotropy of permeability. The findings of this study reveal the importance of understanding the mechanotransduction in the bone, which will help us better assess some bone diseases such as osteoporosis.

## Figures and Tables

**Table 1:** Geometry and degree of representative cases.

	*a*	*b*	*c*	Degree of anisotropy	Degree of elongation	Degree of flatness
Case 1	6.1	6.1	3.35	0.3	0	0.7
Case 2	7.34	7.34	2.32	0.1	0	0.9
Case 3	8.57	3.82	3.82	0.2	0.8	0
Case 4	7.47	4.09	4.09	0.3	0.7	0
Case 5	6.69	6.24	2.99	0.2	0.13	0.77
Case 6	8.36	4	3.74	0.2	0.77	0.12
Reference	5	5	5	1	0	0

**Table 2:** Geometrical and material constants used in the osteon model [20, 21, 23, 35].

Parameter	Description	Value
*E*_r_	Radial drained Young's modulus	15.9 (GPa)
*ν*_r_	Radial drained Poisson's ratio	0.328
*E*_*z*_	Axial drained Young's modulus	20.3 (GPa)
*ν*_*z*_	Axial drained Poisson's ratio	0.25
*M*	Biot's modulus	38 (GPa)
*α*	Biot's effective coefficient	0.132
*ρ*_S_	Solid density	2000 (kg/m^3^)
*ρ*_f_	Fluid density	1000 (kg/m^3^)
*μ*	Dynamic viscosity	10^−3^ (Pa·s)
*R*_*a*_	Inner radius of bone tissue	50 (*μ*m)
*R*_*b*_	Outer radius of bone tissue	150 (*μ*m)
*C*_*p*_	Fluid compressibility	4 × 10^−10^ (1/Pa)

## Data Availability

Data are available from the corresponding author upon reasonable request.

## Nomenclature

PP: Pore pressure
FV: Fluid velocity
FSS: Fluid shear stress
CPUC: Cubic periodic unit cell
*r*_c_: The radius of the bone canaliculi
*r*_o_: The radius of the osteocyte process
*a*_o_: The radius of the pericellular fibers
Δ: The effective spacing of the fibers of the pericellular matrix
*q*: A dimensionless ratio between the radius of the bone canaliculus (*r*_c_) and the osteocyte process (*r*_o_)
*γ*: A dimensionless length ratio between *r*_c_ and the square root of the permeability of a single canaliculus ($\gamma =\text{rc}/\sqrt{k_{p}}$)
*k*_p_: The permeability of the fibre-filled medium in a single canaliculus *k*_p_ = 0.0572*a*_0_^2^(Δ/*a*_0_)^2.377^
*n*_*i*_(*i* = *x*, *y*, *z*): The number of canaliculi passing through each face of the CPUC perpendicular to the local osteocyte axes (*x*, *y*, and *z*), respectively
*a*, *b*, and *c*: Semiaxes of the osteocyte lacunar ellipsoid
*L*: The distance between the two lacunae, which is also the side length of CPUC
*V*_*L*_: The unit volume
*N*_Lac_: The number of the lacunae per unit volume
*N*: The total number of the bone canaliculi *N* around each lacuna; the porosity of the lacuno-canalicular
*L*_c_: The average length of the bone canaliculi
*r*_Lac_: The average radius of lacunae in the radial direction
*S*_*x*_, *S*_*y*_, and *S*_*z*_: The projected surface areas of the lacunar ellipsoid in the *x*, *y*, and *z* orientations, respectively
*K*: The permeability tensor
*E*_r_: Radial drained Young's modulus
EV1: The square of the long half axis
EV2: The square of the middle half axis
EV3: The square of the long half axis
*R*: The reaction
*ν*_r_: Radial drained Poisson's ratio
*E*_*z*_: Axial drained Young's modulus
*ν*_*z*_: Axial drained Poisson's ratio
*M*: Biot's modulus
*α*: Biot's effective coefficient
*ρ*_S_: Solid density
*ρ*_f_: Fluid density
*μ*: Dynamic viscosity
*R*_*a*_: Inner radius of bone tissue
*R*_*b*_: Outer radius of bone tissue
*C*_*p*_: Fluid compressibility
*σ*: The total stress tensor
*C*: The drained stiffness tensor
*ε*: The total strain tensor
*ζ*: The variation in fluid content
**t** **r**(): The trace operator
*ρ*: The total density
$\ddot{u}$: The second derivatives of the displacement
**V**: The velocity vector
*d*: The mean pore diameter
*v*_*r*_: The interstitial fluid velocity
*T*: The tortuosity of the flow path
*w*: Harmonic displacement
*C*_*i*_: The concentration of the reactants
*S*: Reaction product
*D*_C_: The convective flux
*D*_d_: The diffusive flux
*D*: The species' diffusion coefficient.

## Supplementary Materials

Supplementary Materials All the data and images were calculated by the finite element model. The model is in a folder named Finite element model. If reviewers and editors want to repeat the experiment or verify the reliability of experimental modal data, they can directly use the finite element model provided by the author. It is worth noting that COMSOL software is used in this study. The folder named Case 1 and Case 2 represent the results of the model under Case 1 and Case 2 boundary conditions, respectively.
