# Supplementary figures and images for: Effects of Osteocyte Shape on Fluid Flow and Fluid Shear Stress of the Loaded Bone (part 2 of 2)
Source: Biomed Res Int. 2022 May 30;2022:3935803. doi: 10.1155/2022/3935803 (PMC9170394; doi:10.1155/2022/3935803)

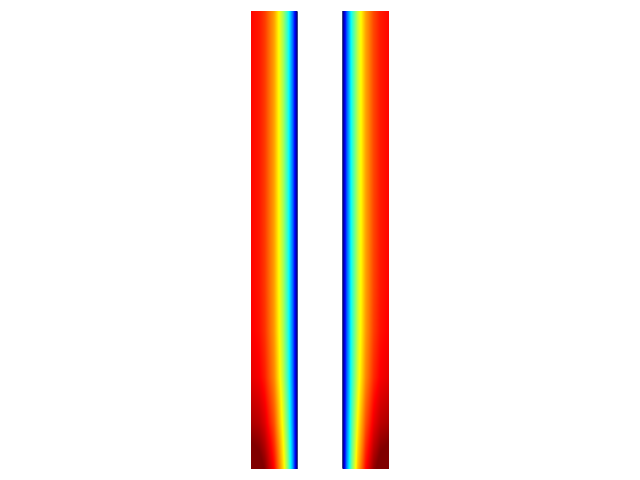

Supplement: Supplementary Materials — All the data and images were calculated by the finite element model. The model is in a folder named Finite element model. If reviewers and editors want to repeat the experiment or verify the reliability of experimental modal data, they can directly use the finite element model provided by the author. It is worth noting that COMSOL software is used in this study. The folder named Case 1 and Case 2 represent the results of the model under Case 1 and Case 2 boundary conditions, respectively. [file 3935803.f1.zip › 3935803.f1/BC1/PP/k6-xz-__ .png]

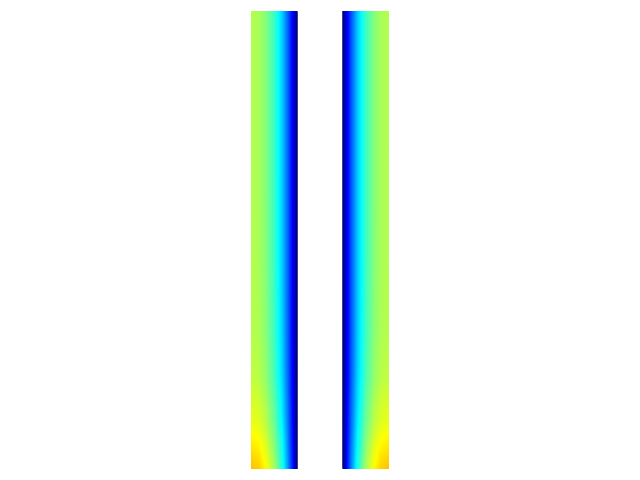

Supplement: Supplementary Materials — All the data and images were calculated by the finite element model. The model is in a folder named Finite element model. If reviewers and editors want to repeat the experiment or verify the reliability of experimental modal data, they can directly use the finite element model provided by the author. It is worth noting that COMSOL software is used in this study. The folder named Case 1 and Case 2 represent the results of the model under Case 1 and Case 2 boundary conditions, respectively. [file 3935803.f1.zip › 3935803.f1/BC1/PP/k6-yz-__ .png]

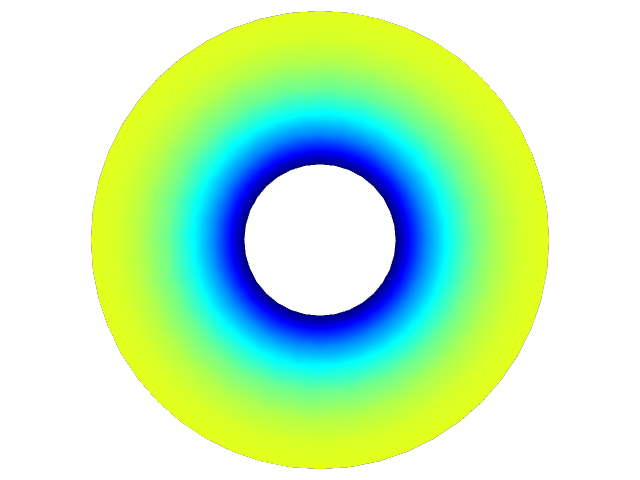

Supplement: Supplementary Materials — All the data and images were calculated by the finite element model. The model is in a folder named Finite element model. If reviewers and editors want to repeat the experiment or verify the reliability of experimental modal data, they can directly use the finite element model provided by the author. It is worth noting that COMSOL software is used in this study. The folder named Case 1 and Case 2 represent the results of the model under Case 1 and Case 2 boundary conditions, respectively. [file 3935803.f1.zip › 3935803.f1/BC1/PP/k7-x-__ .png]

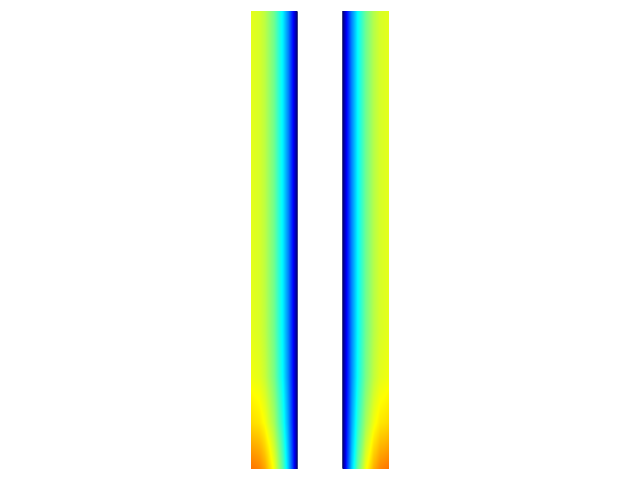

Supplement: Supplementary Materials — All the data and images were calculated by the finite element model. The model is in a folder named Finite element model. If reviewers and editors want to repeat the experiment or verify the reliability of experimental modal data, they can directly use the finite element model provided by the author. It is worth noting that COMSOL software is used in this study. The folder named Case 1 and Case 2 represent the results of the model under Case 1 and Case 2 boundary conditions, respectively. [file 3935803.f1.zip › 3935803.f1/BC1/PP/k7-xz-__ .png]

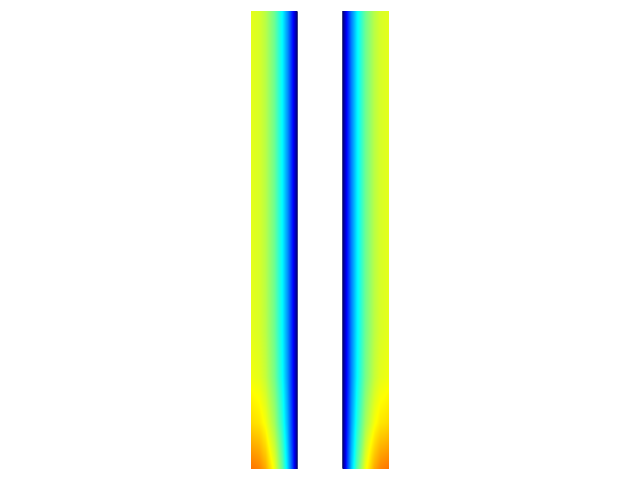

Supplement: Supplementary Materials — All the data and images were calculated by the finite element model. The model is in a folder named Finite element model. If reviewers and editors want to repeat the experiment or verify the reliability of experimental modal data, they can directly use the finite element model provided by the author. It is worth noting that COMSOL software is used in this study. The folder named Case 1 and Case 2 represent the results of the model under Case 1 and Case 2 boundary conditions, respectively. [file 3935803.f1.zip › 3935803.f1/BC1/PP/k7-yz-__ .png]

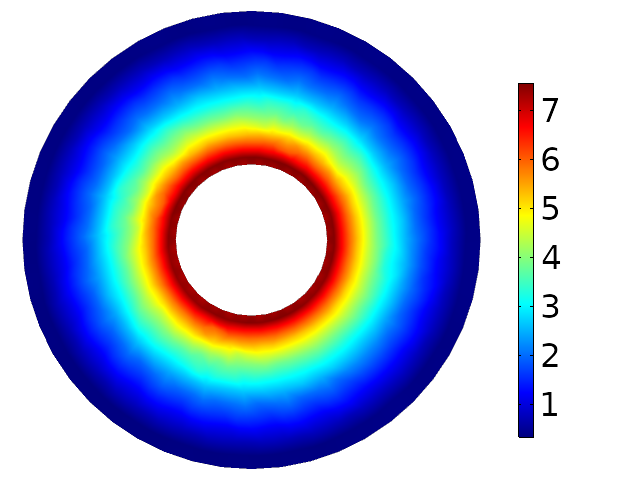

Supplement: Supplementary Materials — All the data and images were calculated by the finite element model. The model is in a folder named Finite element model. If reviewers and editors want to repeat the experiment or verify the reliability of experimental modal data, they can directly use the finite element model provided by the author. It is worth noting that COMSOL software is used in this study. The folder named Case 1 and Case 2 represent the results of the model under Case 1 and Case 2 boundary conditions, respectively. [file 3935803.f1.zip › 3935803.f1/BC2/FSS/____/__.png]

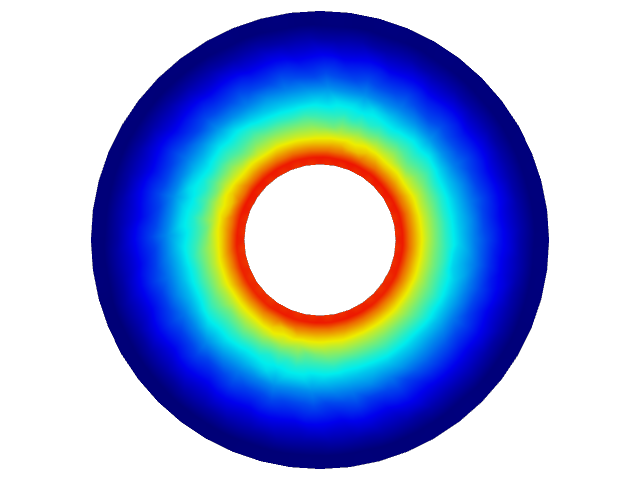

Supplement: Supplementary Materials — All the data and images were calculated by the finite element model. The model is in a folder named Finite element model. If reviewers and editors want to repeat the experiment or verify the reliability of experimental modal data, they can directly use the finite element model provided by the author. It is worth noting that COMSOL software is used in this study. The folder named Case 1 and Case 2 represent the results of the model under Case 1 and Case 2 boundary conditions, respectively. [file 3935803.f1.zip › 3935803.f1/BC2/FSS/____/k1___.png]

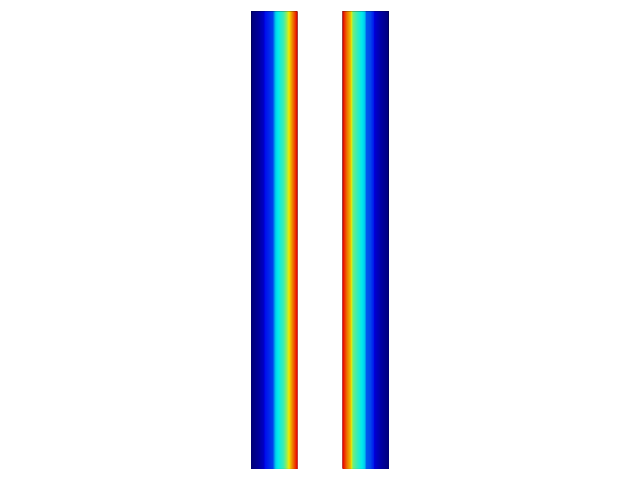

Supplement: Supplementary Materials — All the data and images were calculated by the finite element model. The model is in a folder named Finite element model. If reviewers and editors want to repeat the experiment or verify the reliability of experimental modal data, they can directly use the finite element model provided by the author. It is worth noting that COMSOL software is used in this study. The folder named Case 1 and Case 2 represent the results of the model under Case 1 and Case 2 boundary conditions, respectively. [file 3935803.f1.zip › 3935803.f1/BC2/FSS/____/k1___xz.png]

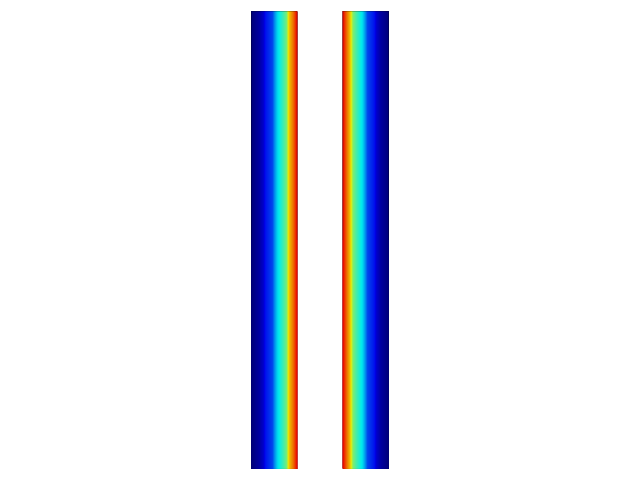

Supplement: Supplementary Materials — All the data and images were calculated by the finite element model. The model is in a folder named Finite element model. If reviewers and editors want to repeat the experiment or verify the reliability of experimental modal data, they can directly use the finite element model provided by the author. It is worth noting that COMSOL software is used in this study. The folder named Case 1 and Case 2 represent the results of the model under Case 1 and Case 2 boundary conditions, respectively. [file 3935803.f1.zip › 3935803.f1/BC2/FSS/____/k1___yz.png]

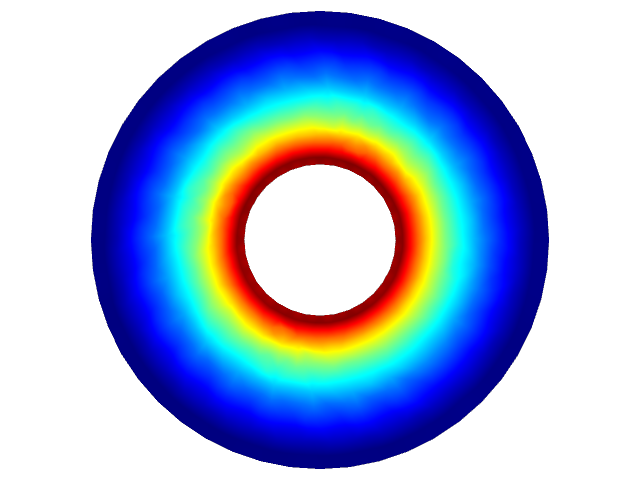

Supplement: Supplementary Materials — All the data and images were calculated by the finite element model. The model is in a folder named Finite element model. If reviewers and editors want to repeat the experiment or verify the reliability of experimental modal data, they can directly use the finite element model provided by the author. It is worth noting that COMSOL software is used in this study. The folder named Case 1 and Case 2 represent the results of the model under Case 1 and Case 2 boundary conditions, respectively. [file 3935803.f1.zip › 3935803.f1/BC2/FSS/____/k2___.png]

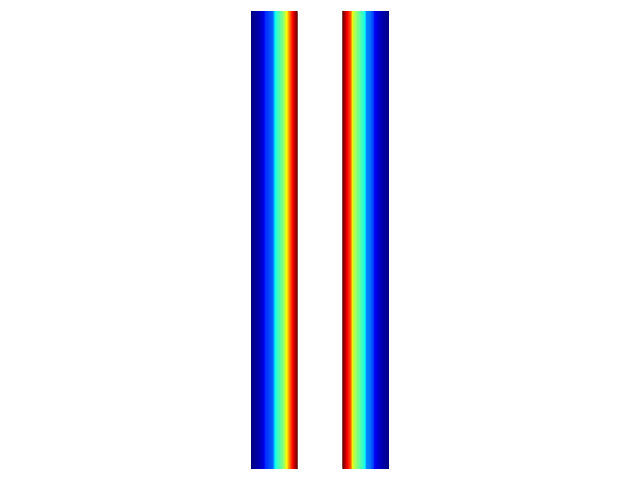

Supplement: Supplementary Materials — All the data and images were calculated by the finite element model. The model is in a folder named Finite element model. If reviewers and editors want to repeat the experiment or verify the reliability of experimental modal data, they can directly use the finite element model provided by the author. It is worth noting that COMSOL software is used in this study. The folder named Case 1 and Case 2 represent the results of the model under Case 1 and Case 2 boundary conditions, respectively. [file 3935803.f1.zip › 3935803.f1/BC2/FSS/____/k2___xz.png]

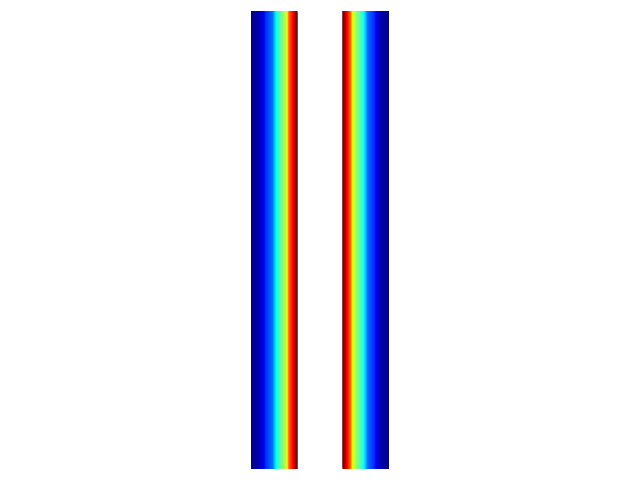

Supplement: Supplementary Materials — All the data and images were calculated by the finite element model. The model is in a folder named Finite element model. If reviewers and editors want to repeat the experiment or verify the reliability of experimental modal data, they can directly use the finite element model provided by the author. It is worth noting that COMSOL software is used in this study. The folder named Case 1 and Case 2 represent the results of the model under Case 1 and Case 2 boundary conditions, respectively. [file 3935803.f1.zip › 3935803.f1/BC2/FSS/____/k2___yz.png]

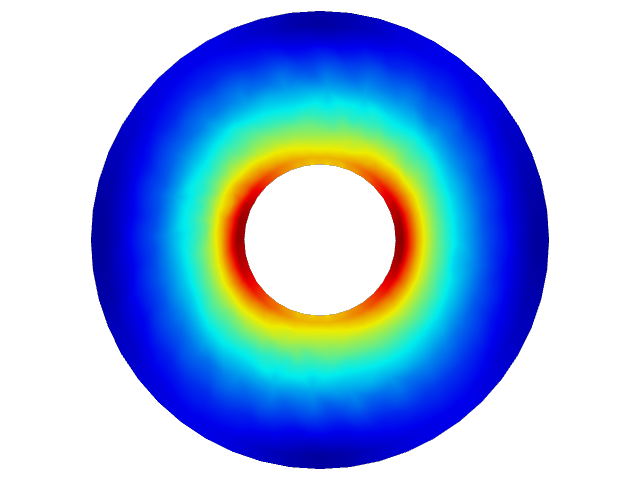

Supplement: Supplementary Materials — All the data and images were calculated by the finite element model. The model is in a folder named Finite element model. If reviewers and editors want to repeat the experiment or verify the reliability of experimental modal data, they can directly use the finite element model provided by the author. It is worth noting that COMSOL software is used in this study. The folder named Case 1 and Case 2 represent the results of the model under Case 1 and Case 2 boundary conditions, respectively. [file 3935803.f1.zip › 3935803.f1/BC2/FSS/____/k3___.png]

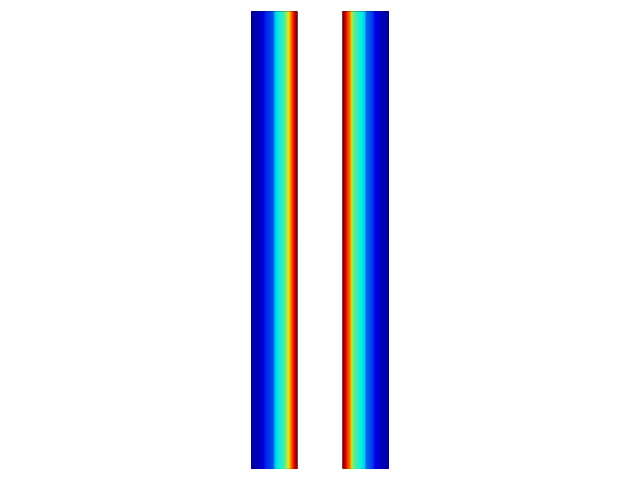

Supplement: Supplementary Materials — All the data and images were calculated by the finite element model. The model is in a folder named Finite element model. If reviewers and editors want to repeat the experiment or verify the reliability of experimental modal data, they can directly use the finite element model provided by the author. It is worth noting that COMSOL software is used in this study. The folder named Case 1 and Case 2 represent the results of the model under Case 1 and Case 2 boundary conditions, respectively. [file 3935803.f1.zip › 3935803.f1/BC2/FSS/____/k3___xz.png]

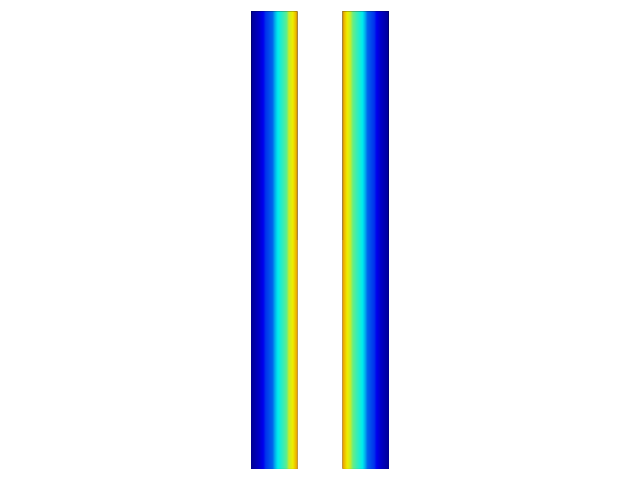

Supplement: Supplementary Materials — All the data and images were calculated by the finite element model. The model is in a folder named Finite element model. If reviewers and editors want to repeat the experiment or verify the reliability of experimental modal data, they can directly use the finite element model provided by the author. It is worth noting that COMSOL software is used in this study. The folder named Case 1 and Case 2 represent the results of the model under Case 1 and Case 2 boundary conditions, respectively. [file 3935803.f1.zip › 3935803.f1/BC2/FSS/____/k3___yz.png]

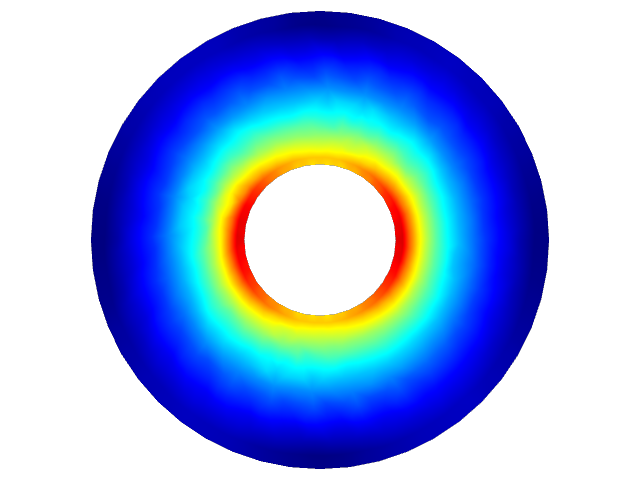

Supplement: Supplementary Materials — All the data and images were calculated by the finite element model. The model is in a folder named Finite element model. If reviewers and editors want to repeat the experiment or verify the reliability of experimental modal data, they can directly use the finite element model provided by the author. It is worth noting that COMSOL software is used in this study. The folder named Case 1 and Case 2 represent the results of the model under Case 1 and Case 2 boundary conditions, respectively. [file 3935803.f1.zip › 3935803.f1/BC2/FSS/____/k4___.png]

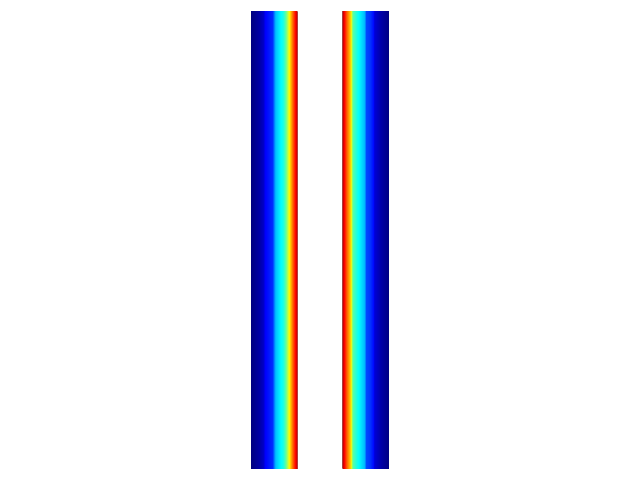

Supplement: Supplementary Materials — All the data and images were calculated by the finite element model. The model is in a folder named Finite element model. If reviewers and editors want to repeat the experiment or verify the reliability of experimental modal data, they can directly use the finite element model provided by the author. It is worth noting that COMSOL software is used in this study. The folder named Case 1 and Case 2 represent the results of the model under Case 1 and Case 2 boundary conditions, respectively. [file 3935803.f1.zip › 3935803.f1/BC2/FSS/____/k4___xz.png]

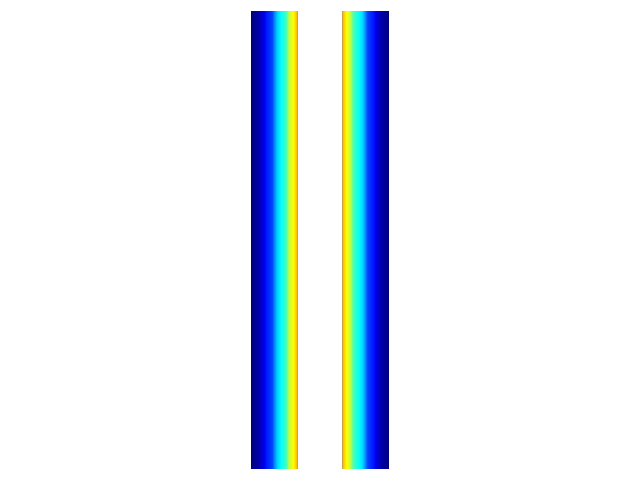

Supplement: Supplementary Materials — All the data and images were calculated by the finite element model. The model is in a folder named Finite element model. If reviewers and editors want to repeat the experiment or verify the reliability of experimental modal data, they can directly use the finite element model provided by the author. It is worth noting that COMSOL software is used in this study. The folder named Case 1 and Case 2 represent the results of the model under Case 1 and Case 2 boundary conditions, respectively. [file 3935803.f1.zip › 3935803.f1/BC2/FSS/____/k4___yz.png]

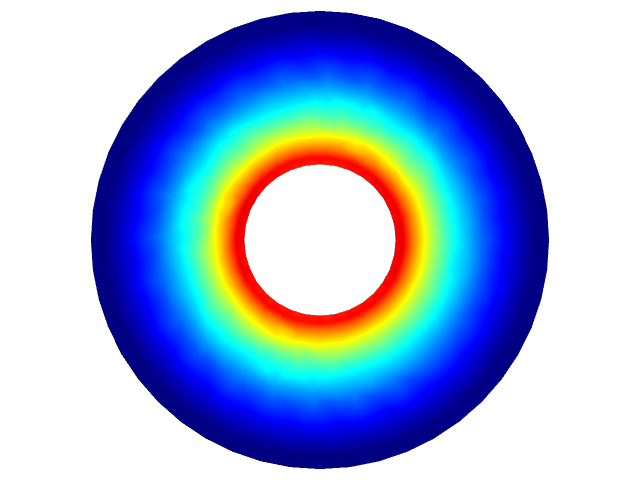

Supplement: Supplementary Materials — All the data and images were calculated by the finite element model. The model is in a folder named Finite element model. If reviewers and editors want to repeat the experiment or verify the reliability of experimental modal data, they can directly use the finite element model provided by the author. It is worth noting that COMSOL software is used in this study. The folder named Case 1 and Case 2 represent the results of the model under Case 1 and Case 2 boundary conditions, respectively. [file 3935803.f1.zip › 3935803.f1/BC2/FSS/____/k5___.png]

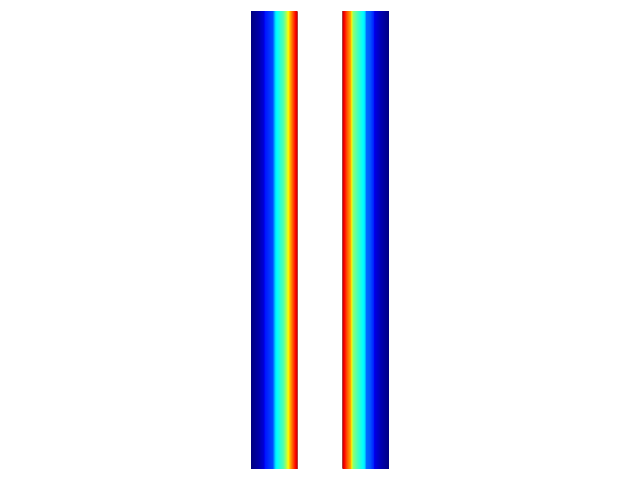

Supplement: Supplementary Materials — All the data and images were calculated by the finite element model. The model is in a folder named Finite element model. If reviewers and editors want to repeat the experiment or verify the reliability of experimental modal data, they can directly use the finite element model provided by the author. It is worth noting that COMSOL software is used in this study. The folder named Case 1 and Case 2 represent the results of the model under Case 1 and Case 2 boundary conditions, respectively. [file 3935803.f1.zip › 3935803.f1/BC2/FSS/____/k5___xz.png]

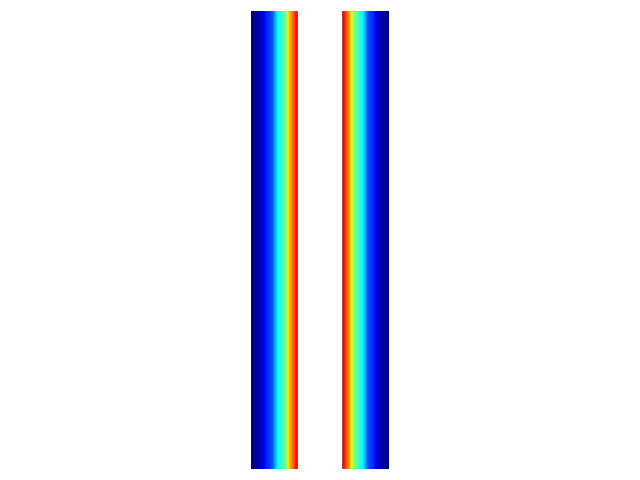

Supplement: Supplementary Materials — All the data and images were calculated by the finite element model. The model is in a folder named Finite element model. If reviewers and editors want to repeat the experiment or verify the reliability of experimental modal data, they can directly use the finite element model provided by the author. It is worth noting that COMSOL software is used in this study. The folder named Case 1 and Case 2 represent the results of the model under Case 1 and Case 2 boundary conditions, respectively. [file 3935803.f1.zip › 3935803.f1/BC2/FSS/____/k5___yz.png]

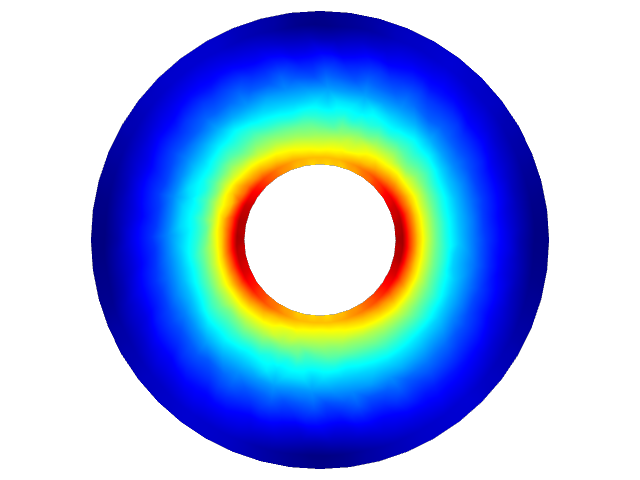

Supplement: Supplementary Materials — All the data and images were calculated by the finite element model. The model is in a folder named Finite element model. If reviewers and editors want to repeat the experiment or verify the reliability of experimental modal data, they can directly use the finite element model provided by the author. It is worth noting that COMSOL software is used in this study. The folder named Case 1 and Case 2 represent the results of the model under Case 1 and Case 2 boundary conditions, respectively. [file 3935803.f1.zip › 3935803.f1/BC2/FSS/____/k6___.png]

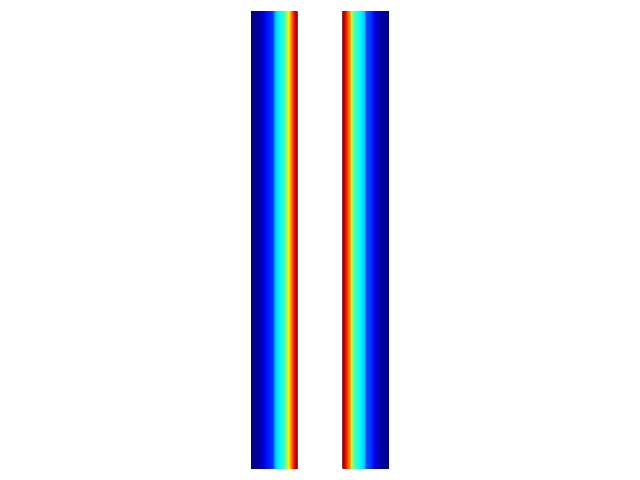

Supplement: Supplementary Materials — All the data and images were calculated by the finite element model. The model is in a folder named Finite element model. If reviewers and editors want to repeat the experiment or verify the reliability of experimental modal data, they can directly use the finite element model provided by the author. It is worth noting that COMSOL software is used in this study. The folder named Case 1 and Case 2 represent the results of the model under Case 1 and Case 2 boundary conditions, respectively. [file 3935803.f1.zip › 3935803.f1/BC2/FSS/____/k6___xz.png]

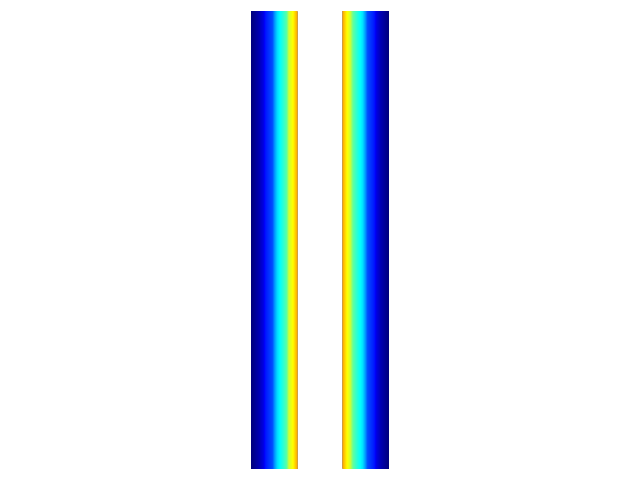

Supplement: Supplementary Materials — All the data and images were calculated by the finite element model. The model is in a folder named Finite element model. If reviewers and editors want to repeat the experiment or verify the reliability of experimental modal data, they can directly use the finite element model provided by the author. It is worth noting that COMSOL software is used in this study. The folder named Case 1 and Case 2 represent the results of the model under Case 1 and Case 2 boundary conditions, respectively. [file 3935803.f1.zip › 3935803.f1/BC2/FSS/____/k6___yz.png]

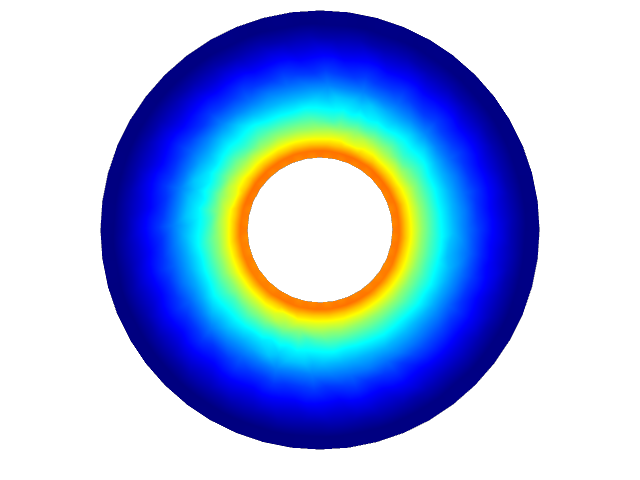

Supplement: Supplementary Materials — All the data and images were calculated by the finite element model. The model is in a folder named Finite element model. If reviewers and editors want to repeat the experiment or verify the reliability of experimental modal data, they can directly use the finite element model provided by the author. It is worth noting that COMSOL software is used in this study. The folder named Case 1 and Case 2 represent the results of the model under Case 1 and Case 2 boundary conditions, respectively. [file 3935803.f1.zip › 3935803.f1/BC2/FSS/____/k7___.png]

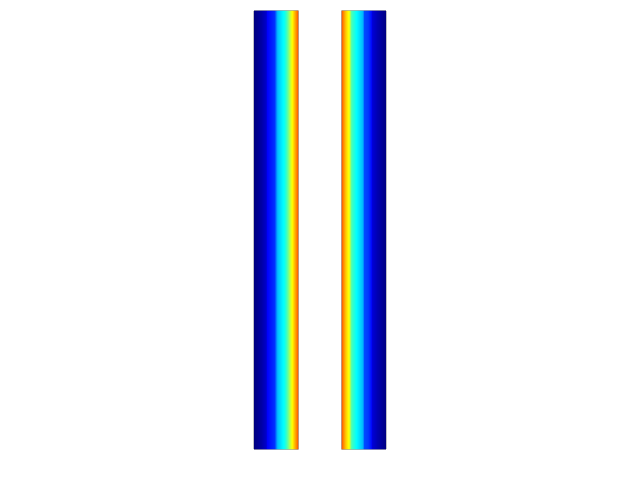

Supplement: Supplementary Materials — All the data and images were calculated by the finite element model. The model is in a folder named Finite element model. If reviewers and editors want to repeat the experiment or verify the reliability of experimental modal data, they can directly use the finite element model provided by the author. It is worth noting that COMSOL software is used in this study. The folder named Case 1 and Case 2 represent the results of the model under Case 1 and Case 2 boundary conditions, respectively. [file 3935803.f1.zip › 3935803.f1/BC2/FSS/____/k7___xz.png]

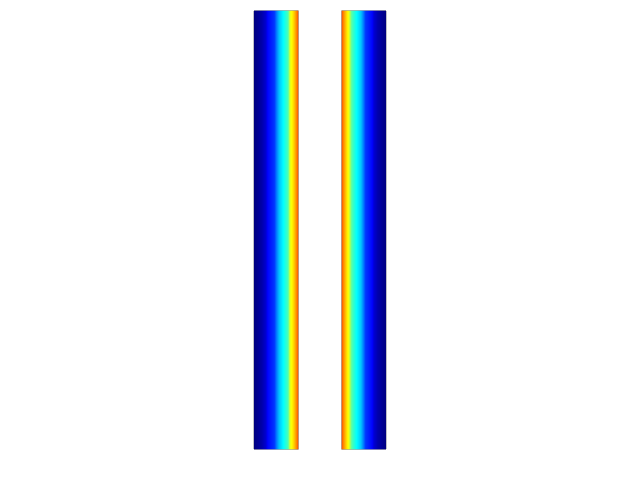

Supplement: Supplementary Materials — All the data and images were calculated by the finite element model. The model is in a folder named Finite element model. If reviewers and editors want to repeat the experiment or verify the reliability of experimental modal data, they can directly use the finite element model provided by the author. It is worth noting that COMSOL software is used in this study. The folder named Case 1 and Case 2 represent the results of the model under Case 1 and Case 2 boundary conditions, respectively. [file 3935803.f1.zip › 3935803.f1/BC2/FSS/____/k7___yz.png]

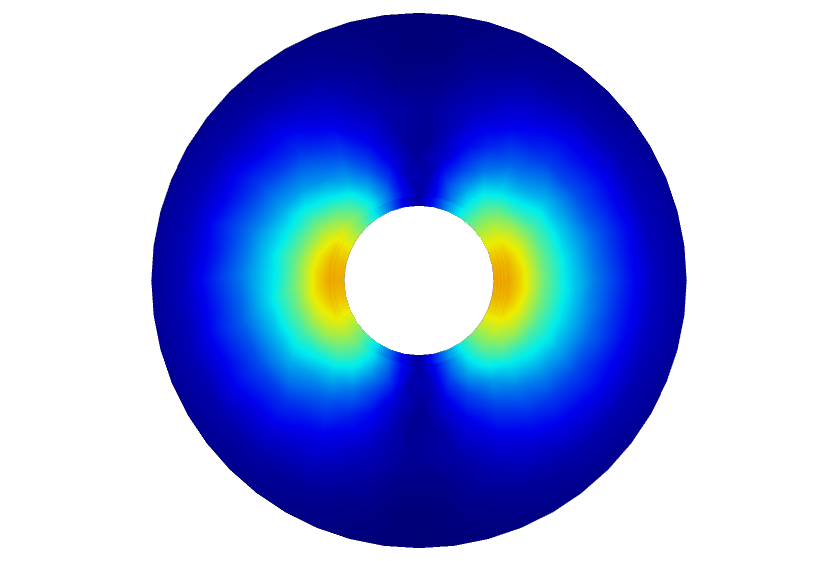

Supplement: Supplementary Materials — All the data and images were calculated by the finite element model. The model is in a folder named Finite element model. If reviewers and editors want to repeat the experiment or verify the reliability of experimental modal data, they can directly use the finite element model provided by the author. It is worth noting that COMSOL software is used in this study. The folder named Case 1 and Case 2 represent the results of the model under Case 1 and Case 2 boundary conditions, respectively. [file 3935803.f1.zip › 3935803.f1/BC2/FSS/k1-x-___ .png]

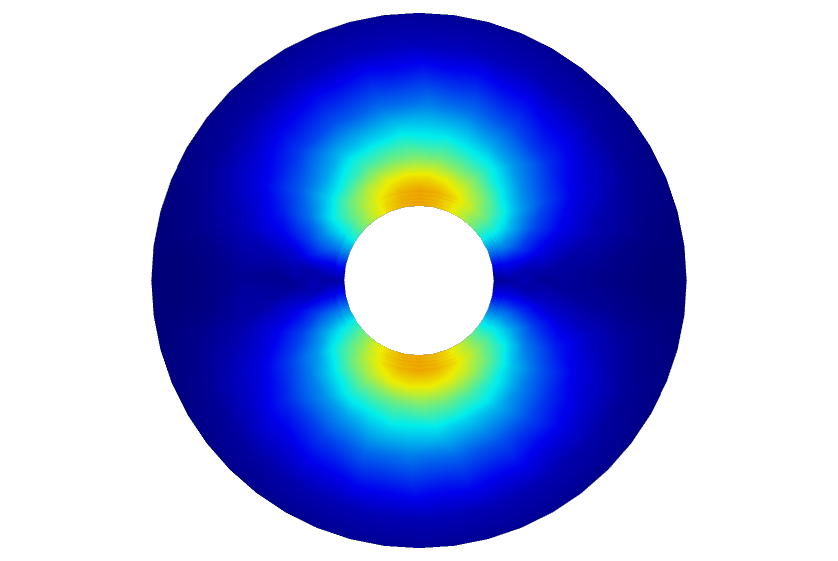

Supplement: Supplementary Materials — All the data and images were calculated by the finite element model. The model is in a folder named Finite element model. If reviewers and editors want to repeat the experiment or verify the reliability of experimental modal data, they can directly use the finite element model provided by the author. It is worth noting that COMSOL software is used in this study. The folder named Case 1 and Case 2 represent the results of the model under Case 1 and Case 2 boundary conditions, respectively. [file 3935803.f1.zip › 3935803.f1/BC2/FSS/k1-y-___ .png]

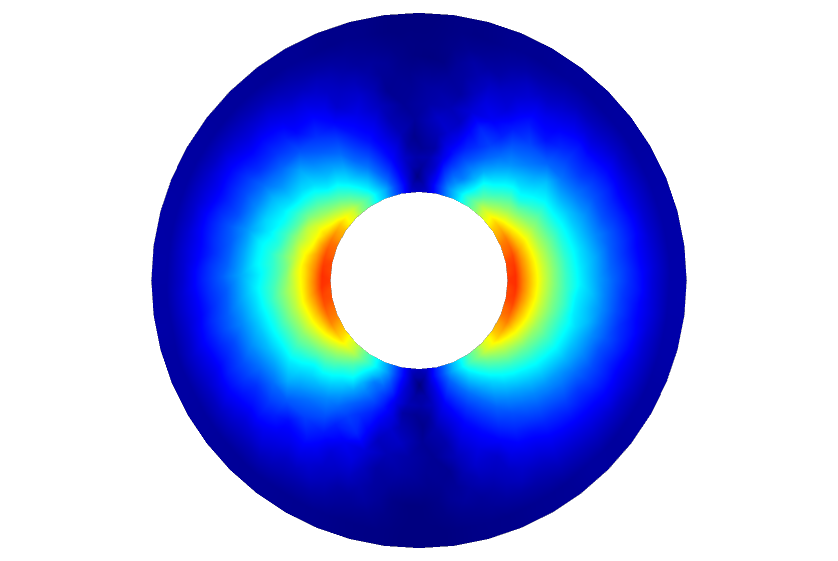

Supplement: Supplementary Materials — All the data and images were calculated by the finite element model. The model is in a folder named Finite element model. If reviewers and editors want to repeat the experiment or verify the reliability of experimental modal data, they can directly use the finite element model provided by the author. It is worth noting that COMSOL software is used in this study. The folder named Case 1 and Case 2 represent the results of the model under Case 1 and Case 2 boundary conditions, respectively. [file 3935803.f1.zip › 3935803.f1/BC2/FSS/k2-x-___ .png]

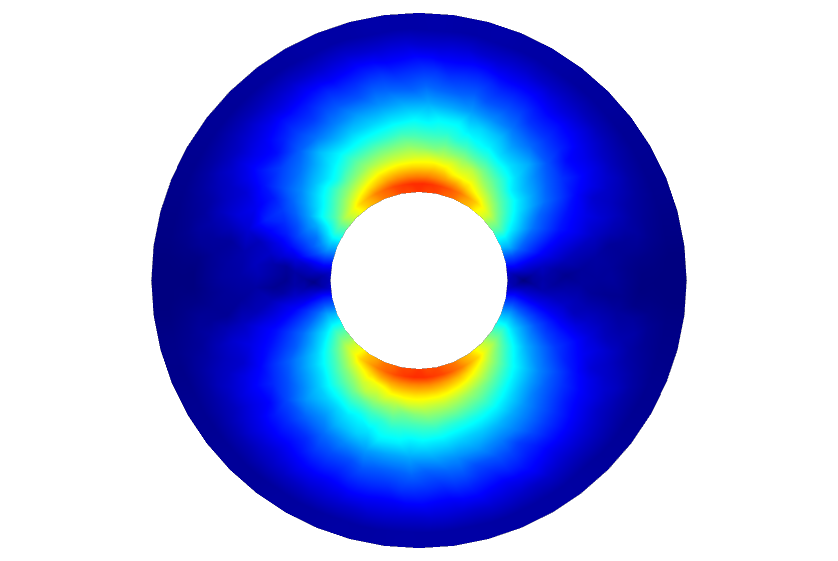

Supplement: Supplementary Materials — All the data and images were calculated by the finite element model. The model is in a folder named Finite element model. If reviewers and editors want to repeat the experiment or verify the reliability of experimental modal data, they can directly use the finite element model provided by the author. It is worth noting that COMSOL software is used in this study. The folder named Case 1 and Case 2 represent the results of the model under Case 1 and Case 2 boundary conditions, respectively. [file 3935803.f1.zip › 3935803.f1/BC2/FSS/k2-y-___ .png]

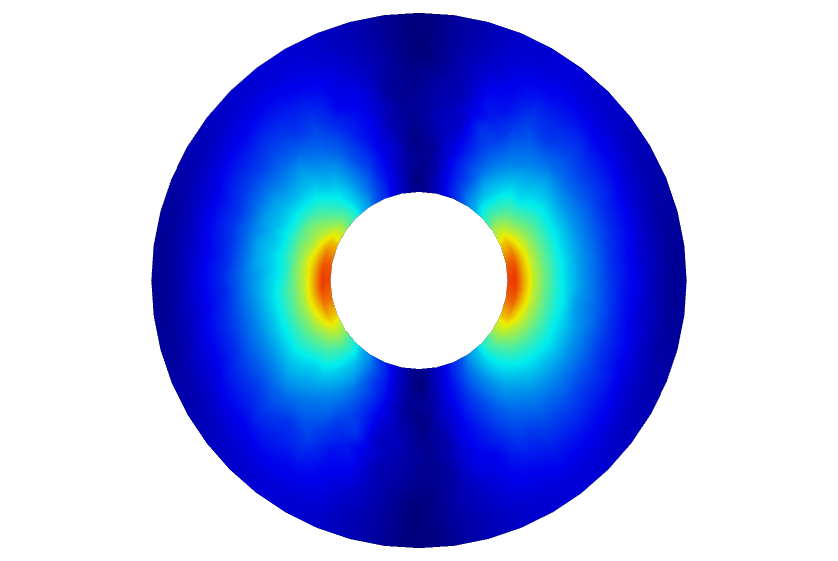

Supplement: Supplementary Materials — All the data and images were calculated by the finite element model. The model is in a folder named Finite element model. If reviewers and editors want to repeat the experiment or verify the reliability of experimental modal data, they can directly use the finite element model provided by the author. It is worth noting that COMSOL software is used in this study. The folder named Case 1 and Case 2 represent the results of the model under Case 1 and Case 2 boundary conditions, respectively. [file 3935803.f1.zip › 3935803.f1/BC2/FSS/k3-x-___ .png]

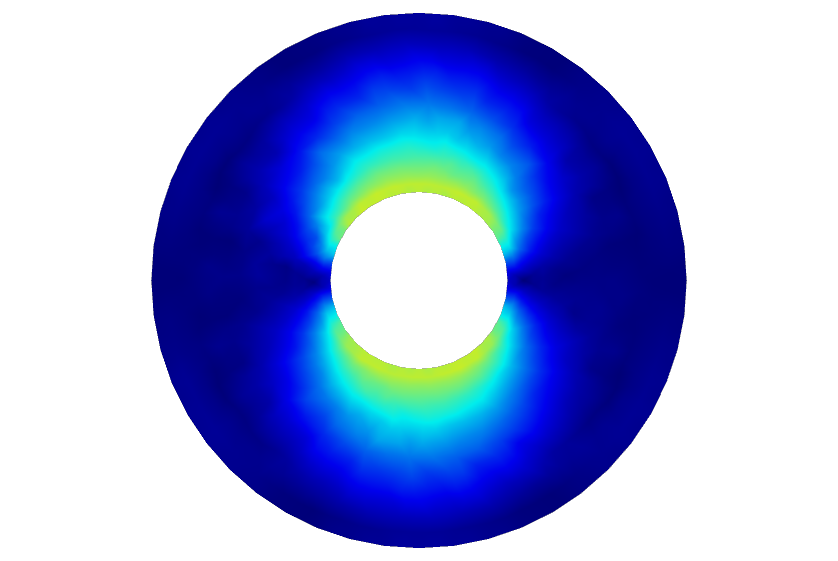

Supplement: Supplementary Materials — All the data and images were calculated by the finite element model. The model is in a folder named Finite element model. If reviewers and editors want to repeat the experiment or verify the reliability of experimental modal data, they can directly use the finite element model provided by the author. It is worth noting that COMSOL software is used in this study. The folder named Case 1 and Case 2 represent the results of the model under Case 1 and Case 2 boundary conditions, respectively. [file 3935803.f1.zip › 3935803.f1/BC2/FSS/k3-y-___ .png]

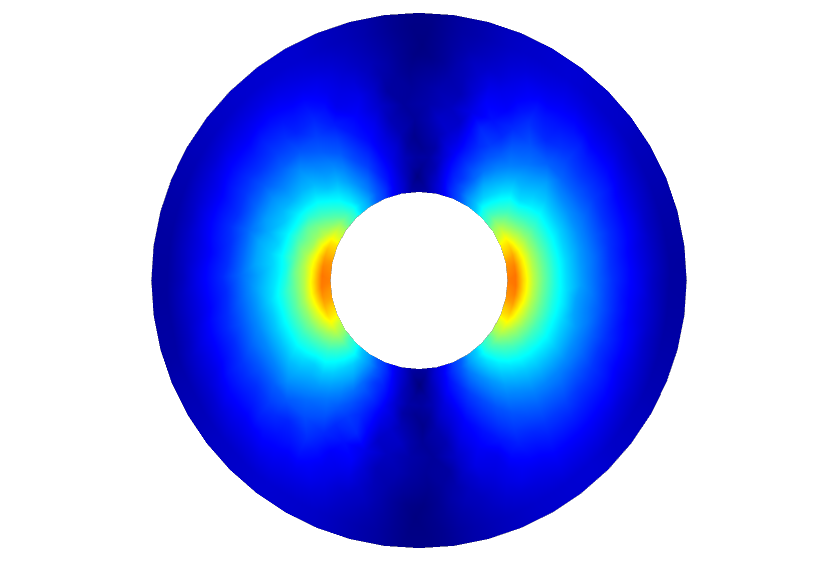

Supplement: Supplementary Materials — All the data and images were calculated by the finite element model. The model is in a folder named Finite element model. If reviewers and editors want to repeat the experiment or verify the reliability of experimental modal data, they can directly use the finite element model provided by the author. It is worth noting that COMSOL software is used in this study. The folder named Case 1 and Case 2 represent the results of the model under Case 1 and Case 2 boundary conditions, respectively. [file 3935803.f1.zip › 3935803.f1/BC2/FSS/k4-x-___ .png]

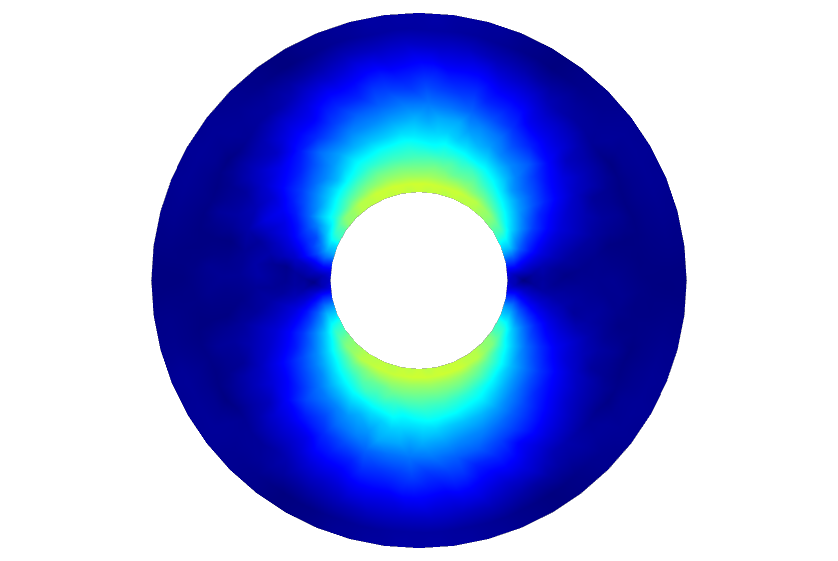

Supplement: Supplementary Materials — All the data and images were calculated by the finite element model. The model is in a folder named Finite element model. If reviewers and editors want to repeat the experiment or verify the reliability of experimental modal data, they can directly use the finite element model provided by the author. It is worth noting that COMSOL software is used in this study. The folder named Case 1 and Case 2 represent the results of the model under Case 1 and Case 2 boundary conditions, respectively. [file 3935803.f1.zip › 3935803.f1/BC2/FSS/k4-y-___ .png]

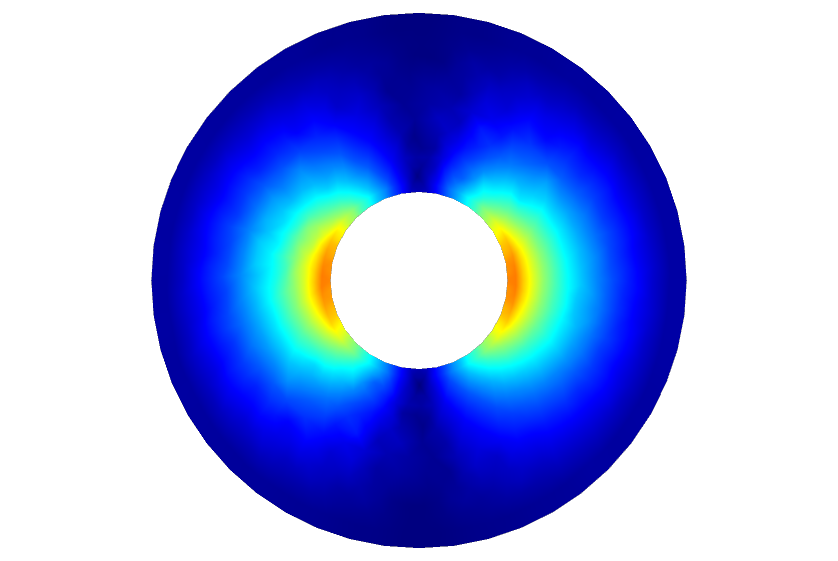

Supplement: Supplementary Materials — All the data and images were calculated by the finite element model. The model is in a folder named Finite element model. If reviewers and editors want to repeat the experiment or verify the reliability of experimental modal data, they can directly use the finite element model provided by the author. It is worth noting that COMSOL software is used in this study. The folder named Case 1 and Case 2 represent the results of the model under Case 1 and Case 2 boundary conditions, respectively. [file 3935803.f1.zip › 3935803.f1/BC2/FSS/k5-x-___ .png]

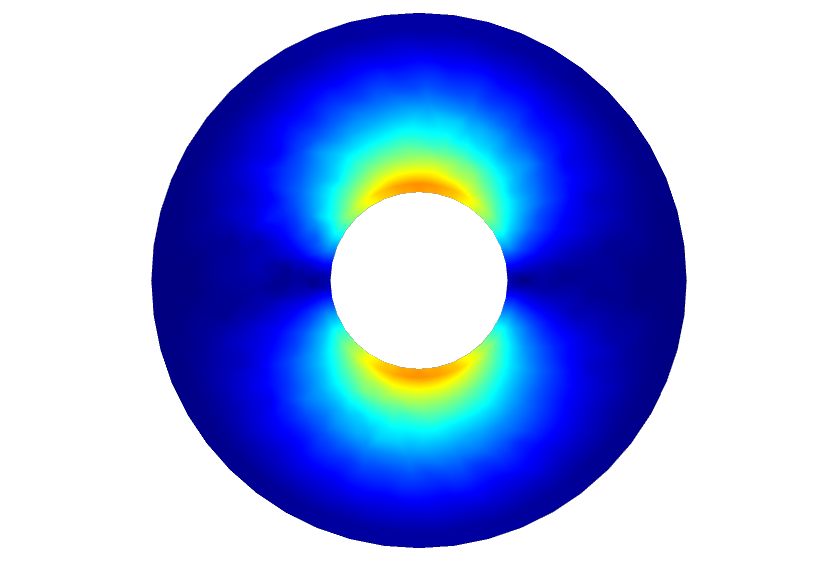

Supplement: Supplementary Materials — All the data and images were calculated by the finite element model. The model is in a folder named Finite element model. If reviewers and editors want to repeat the experiment or verify the reliability of experimental modal data, they can directly use the finite element model provided by the author. It is worth noting that COMSOL software is used in this study. The folder named Case 1 and Case 2 represent the results of the model under Case 1 and Case 2 boundary conditions, respectively. [file 3935803.f1.zip › 3935803.f1/BC2/FSS/k5-y-___ .png]

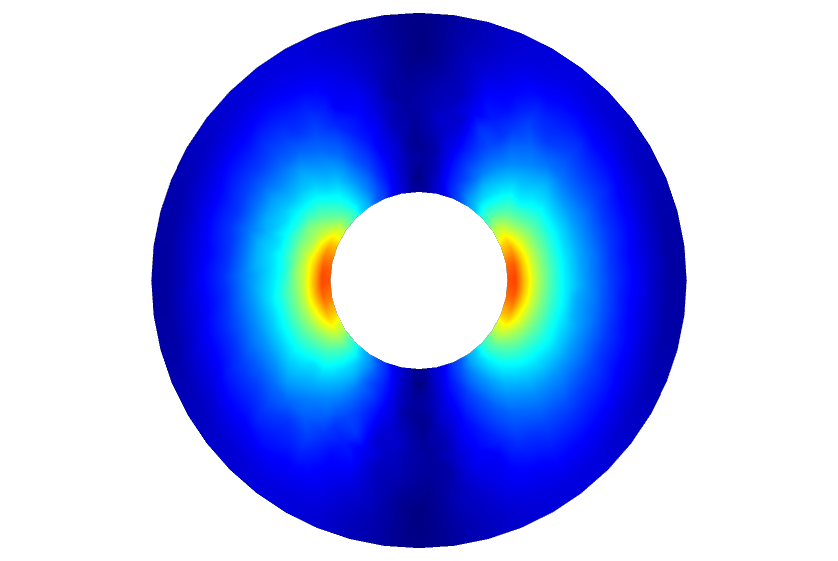

Supplement: Supplementary Materials — All the data and images were calculated by the finite element model. The model is in a folder named Finite element model. If reviewers and editors want to repeat the experiment or verify the reliability of experimental modal data, they can directly use the finite element model provided by the author. It is worth noting that COMSOL software is used in this study. The folder named Case 1 and Case 2 represent the results of the model under Case 1 and Case 2 boundary conditions, respectively. [file 3935803.f1.zip › 3935803.f1/BC2/FSS/k6-x-___ .png]

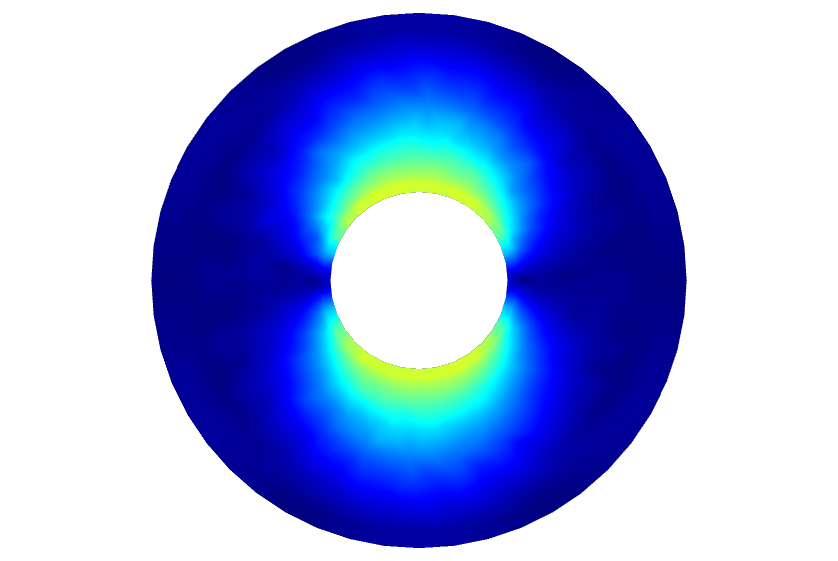

Supplement: Supplementary Materials — All the data and images were calculated by the finite element model. The model is in a folder named Finite element model. If reviewers and editors want to repeat the experiment or verify the reliability of experimental modal data, they can directly use the finite element model provided by the author. It is worth noting that COMSOL software is used in this study. The folder named Case 1 and Case 2 represent the results of the model under Case 1 and Case 2 boundary conditions, respectively. [file 3935803.f1.zip › 3935803.f1/BC2/FSS/k6-y-___ .png]

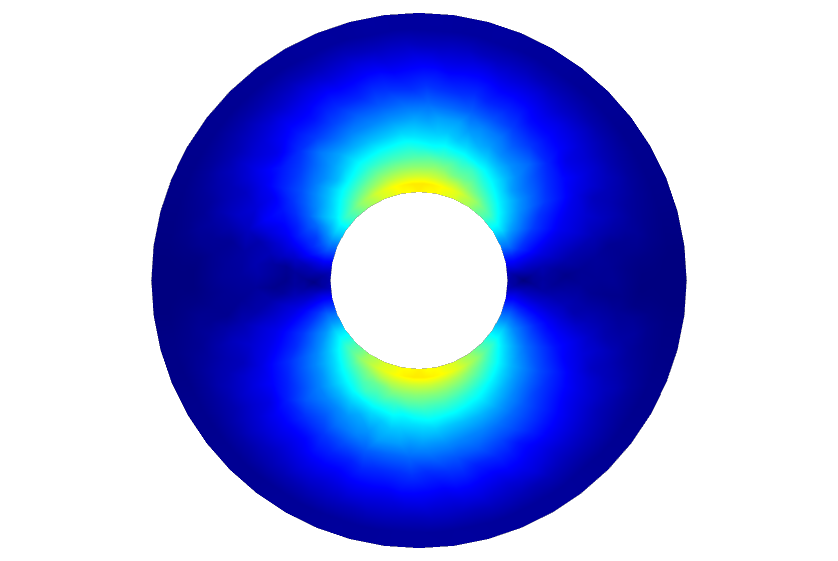

Supplement: Supplementary Materials — All the data and images were calculated by the finite element model. The model is in a folder named Finite element model. If reviewers and editors want to repeat the experiment or verify the reliability of experimental modal data, they can directly use the finite element model provided by the author. It is worth noting that COMSOL software is used in this study. The folder named Case 1 and Case 2 represent the results of the model under Case 1 and Case 2 boundary conditions, respectively. [file 3935803.f1.zip › 3935803.f1/BC2/FSS/k7-x-___ .png]

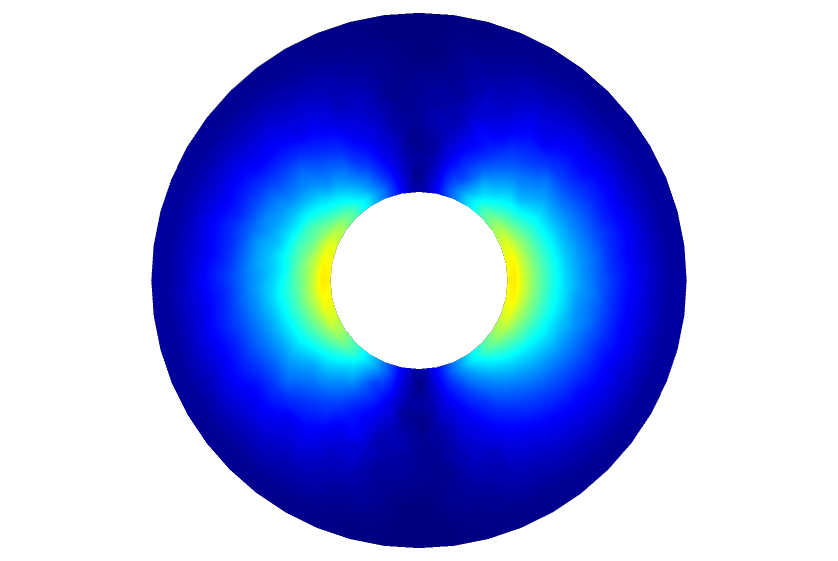

Supplement: Supplementary Materials — All the data and images were calculated by the finite element model. The model is in a folder named Finite element model. If reviewers and editors want to repeat the experiment or verify the reliability of experimental modal data, they can directly use the finite element model provided by the author. It is worth noting that COMSOL software is used in this study. The folder named Case 1 and Case 2 represent the results of the model under Case 1 and Case 2 boundary conditions, respectively. [file 3935803.f1.zip › 3935803.f1/BC2/FSS/k7-y-___ .png]

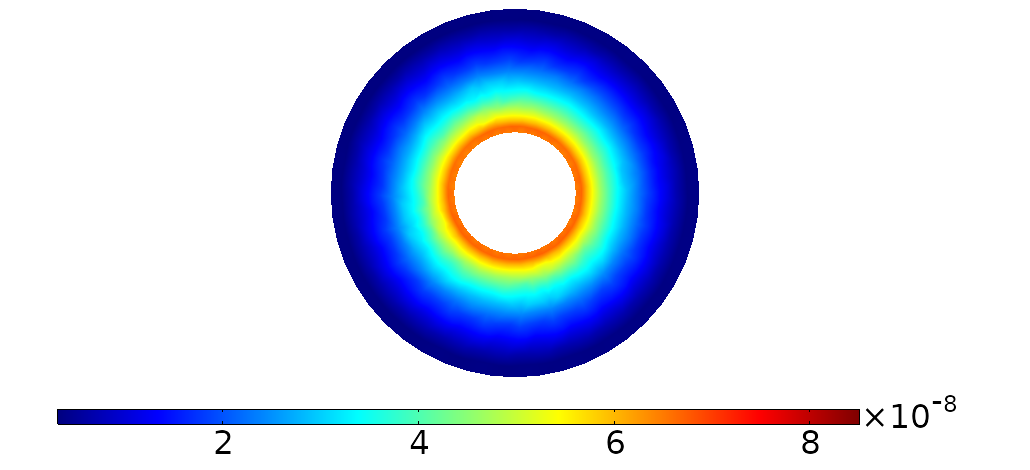

Supplement: Supplementary Materials — All the data and images were calculated by the finite element model. The model is in a folder named Finite element model. If reviewers and editors want to repeat the experiment or verify the reliability of experimental modal data, they can directly use the finite element model provided by the author. It is worth noting that COMSOL software is used in this study. The folder named Case 1 and Case 2 represent the results of the model under Case 1 and Case 2 boundary conditions, respectively. [file 3935803.f1.zip › 3935803.f1/BC2/FV/____.png]

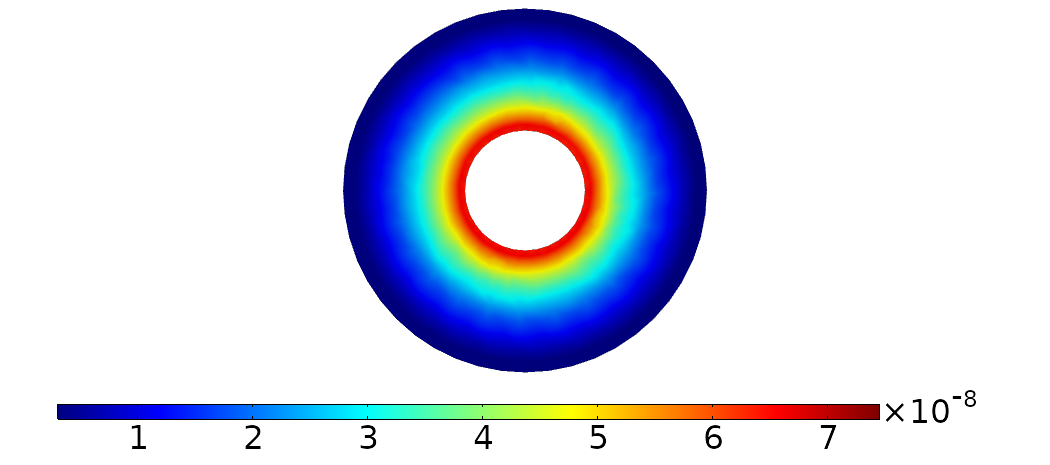

Supplement: Supplementary Materials — All the data and images were calculated by the finite element model. The model is in a folder named Finite element model. If reviewers and editors want to repeat the experiment or verify the reliability of experimental modal data, they can directly use the finite element model provided by the author. It is worth noting that COMSOL software is used in this study. The folder named Case 1 and Case 2 represent the results of the model under Case 1 and Case 2 boundary conditions, respectively. [file 3935803.f1.zip › 3935803.f1/BC2/FV/__z .png]

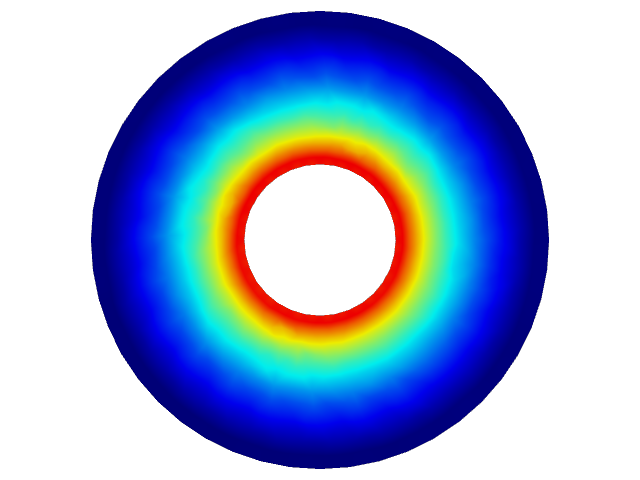

Supplement: Supplementary Materials — All the data and images were calculated by the finite element model. The model is in a folder named Finite element model. If reviewers and editors want to repeat the experiment or verify the reliability of experimental modal data, they can directly use the finite element model provided by the author. It is worth noting that COMSOL software is used in this study. The folder named Case 1 and Case 2 represent the results of the model under Case 1 and Case 2 boundary conditions, respectively. [file 3935803.f1.zip › 3935803.f1/BC2/FV/k1__ .png]

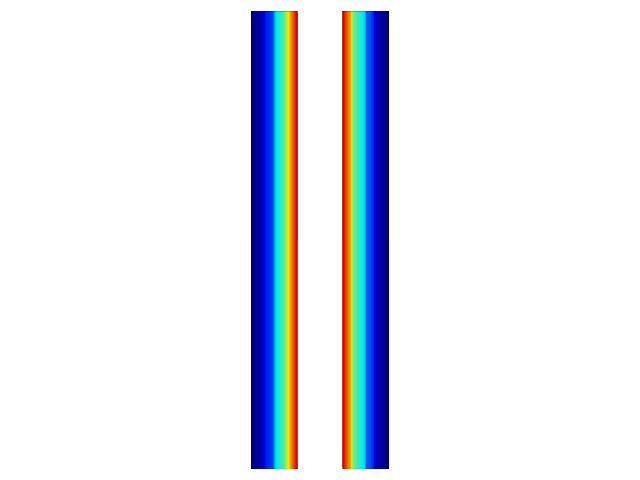

Supplement: Supplementary Materials — All the data and images were calculated by the finite element model. The model is in a folder named Finite element model. If reviewers and editors want to repeat the experiment or verify the reliability of experimental modal data, they can directly use the finite element model provided by the author. It is worth noting that COMSOL software is used in this study. The folder named Case 1 and Case 2 represent the results of the model under Case 1 and Case 2 boundary conditions, respectively. [file 3935803.f1.zip › 3935803.f1/BC2/FV/k1__xz .png]

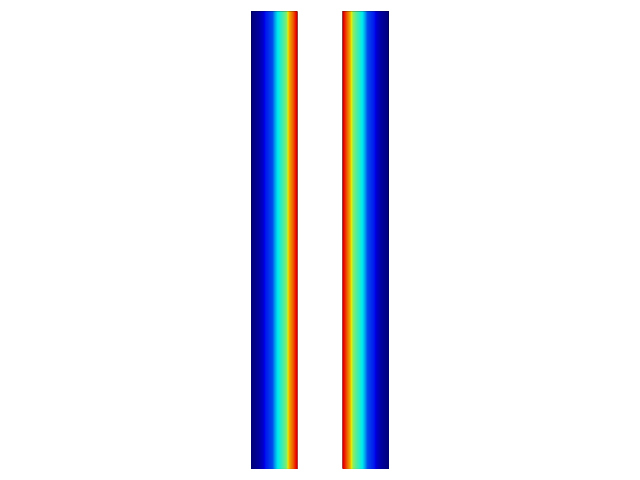

Supplement: Supplementary Materials — All the data and images were calculated by the finite element model. The model is in a folder named Finite element model. If reviewers and editors want to repeat the experiment or verify the reliability of experimental modal data, they can directly use the finite element model provided by the author. It is worth noting that COMSOL software is used in this study. The folder named Case 1 and Case 2 represent the results of the model under Case 1 and Case 2 boundary conditions, respectively. [file 3935803.f1.zip › 3935803.f1/BC2/FV/k1__yz .png]

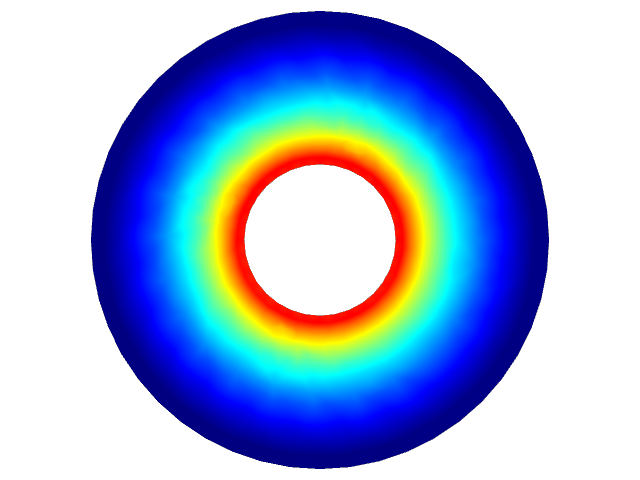

Supplement: Supplementary Materials — All the data and images were calculated by the finite element model. The model is in a folder named Finite element model. If reviewers and editors want to repeat the experiment or verify the reliability of experimental modal data, they can directly use the finite element model provided by the author. It is worth noting that COMSOL software is used in this study. The folder named Case 1 and Case 2 represent the results of the model under Case 1 and Case 2 boundary conditions, respectively. [file 3935803.f1.zip › 3935803.f1/BC2/FV/k2-__ .png]

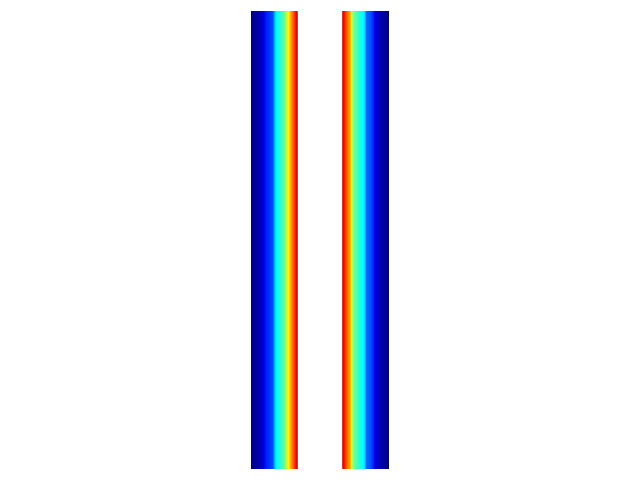

Supplement: Supplementary Materials — All the data and images were calculated by the finite element model. The model is in a folder named Finite element model. If reviewers and editors want to repeat the experiment or verify the reliability of experimental modal data, they can directly use the finite element model provided by the author. It is worth noting that COMSOL software is used in this study. The folder named Case 1 and Case 2 represent the results of the model under Case 1 and Case 2 boundary conditions, respectively. [file 3935803.f1.zip › 3935803.f1/BC2/FV/k2-__xz .png]

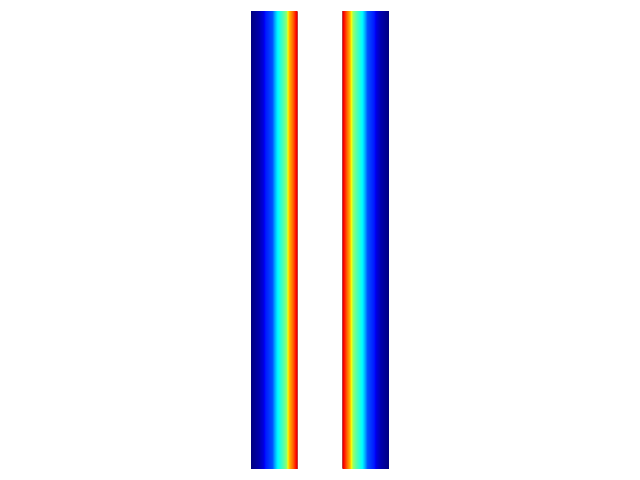

Supplement: Supplementary Materials — All the data and images were calculated by the finite element model. The model is in a folder named Finite element model. If reviewers and editors want to repeat the experiment or verify the reliability of experimental modal data, they can directly use the finite element model provided by the author. It is worth noting that COMSOL software is used in this study. The folder named Case 1 and Case 2 represent the results of the model under Case 1 and Case 2 boundary conditions, respectively. [file 3935803.f1.zip › 3935803.f1/BC2/FV/k2-__yz .png]

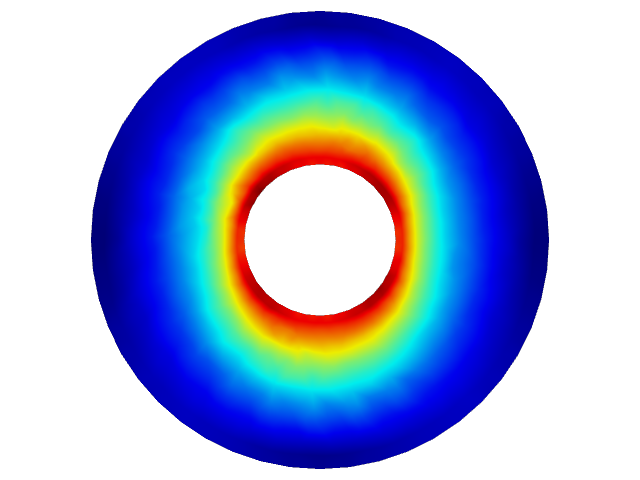

Supplement: Supplementary Materials — All the data and images were calculated by the finite element model. The model is in a folder named Finite element model. If reviewers and editors want to repeat the experiment or verify the reliability of experimental modal data, they can directly use the finite element model provided by the author. It is worth noting that COMSOL software is used in this study. The folder named Case 1 and Case 2 represent the results of the model under Case 1 and Case 2 boundary conditions, respectively. [file 3935803.f1.zip › 3935803.f1/BC2/FV/k3-__ .png]

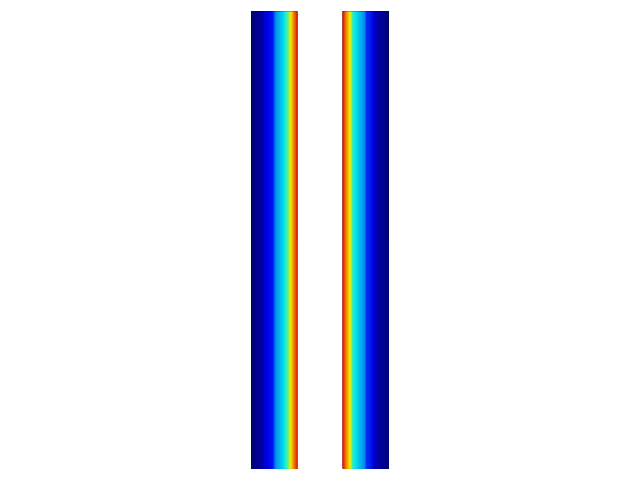

Supplement: Supplementary Materials — All the data and images were calculated by the finite element model. The model is in a folder named Finite element model. If reviewers and editors want to repeat the experiment or verify the reliability of experimental modal data, they can directly use the finite element model provided by the author. It is worth noting that COMSOL software is used in this study. The folder named Case 1 and Case 2 represent the results of the model under Case 1 and Case 2 boundary conditions, respectively. [file 3935803.f1.zip › 3935803.f1/BC2/FV/k3-__xz .png]

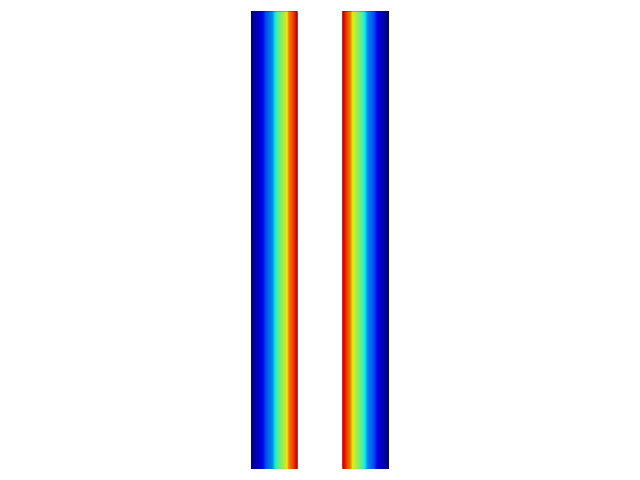

Supplement: Supplementary Materials — All the data and images were calculated by the finite element model. The model is in a folder named Finite element model. If reviewers and editors want to repeat the experiment or verify the reliability of experimental modal data, they can directly use the finite element model provided by the author. It is worth noting that COMSOL software is used in this study. The folder named Case 1 and Case 2 represent the results of the model under Case 1 and Case 2 boundary conditions, respectively. [file 3935803.f1.zip › 3935803.f1/BC2/FV/k3-__yz .png]

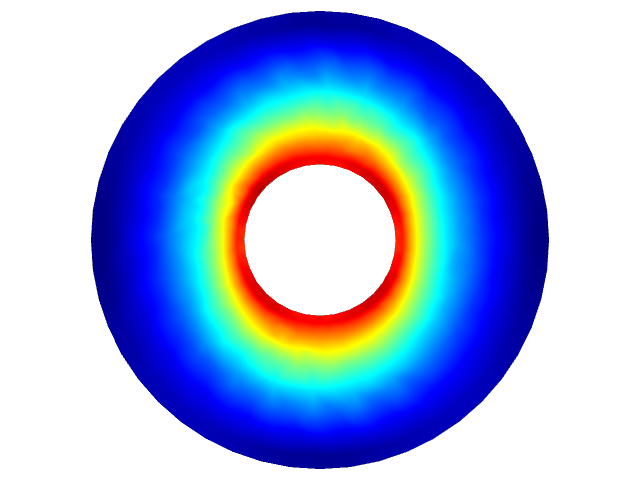

Supplement: Supplementary Materials — All the data and images were calculated by the finite element model. The model is in a folder named Finite element model. If reviewers and editors want to repeat the experiment or verify the reliability of experimental modal data, they can directly use the finite element model provided by the author. It is worth noting that COMSOL software is used in this study. The folder named Case 1 and Case 2 represent the results of the model under Case 1 and Case 2 boundary conditions, respectively. [file 3935803.f1.zip › 3935803.f1/BC2/FV/k4-__ .png]

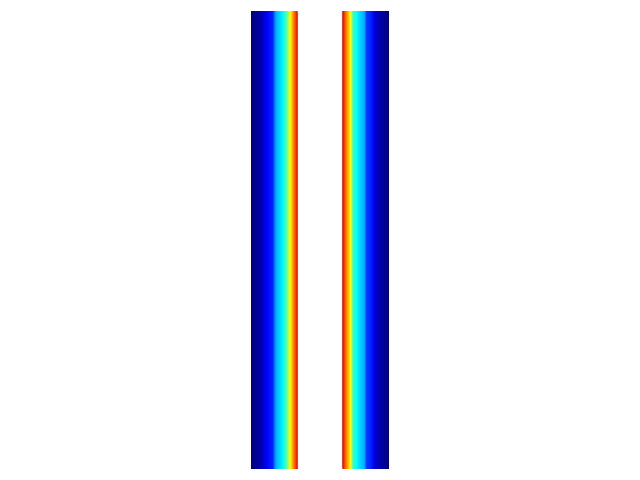

Supplement: Supplementary Materials — All the data and images were calculated by the finite element model. The model is in a folder named Finite element model. If reviewers and editors want to repeat the experiment or verify the reliability of experimental modal data, they can directly use the finite element model provided by the author. It is worth noting that COMSOL software is used in this study. The folder named Case 1 and Case 2 represent the results of the model under Case 1 and Case 2 boundary conditions, respectively. [file 3935803.f1.zip › 3935803.f1/BC2/FV/k4-__xz .png]

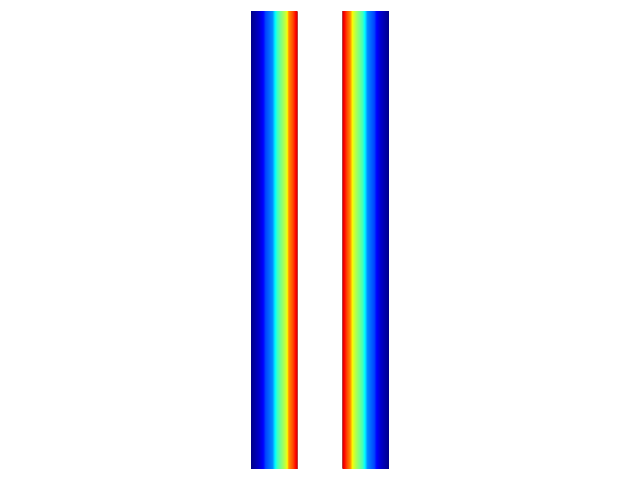

Supplement: Supplementary Materials — All the data and images were calculated by the finite element model. The model is in a folder named Finite element model. If reviewers and editors want to repeat the experiment or verify the reliability of experimental modal data, they can directly use the finite element model provided by the author. It is worth noting that COMSOL software is used in this study. The folder named Case 1 and Case 2 represent the results of the model under Case 1 and Case 2 boundary conditions, respectively. [file 3935803.f1.zip › 3935803.f1/BC2/FV/k4-__yz .png]

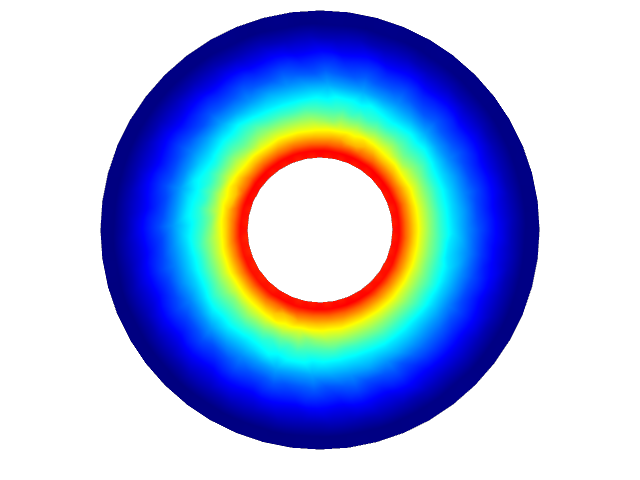

Supplement: Supplementary Materials — All the data and images were calculated by the finite element model. The model is in a folder named Finite element model. If reviewers and editors want to repeat the experiment or verify the reliability of experimental modal data, they can directly use the finite element model provided by the author. It is worth noting that COMSOL software is used in this study. The folder named Case 1 and Case 2 represent the results of the model under Case 1 and Case 2 boundary conditions, respectively. [file 3935803.f1.zip › 3935803.f1/BC2/FV/k5-__ .png]

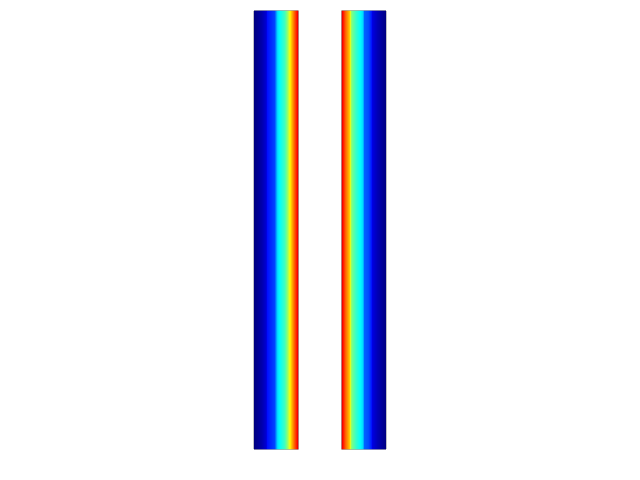

Supplement: Supplementary Materials — All the data and images were calculated by the finite element model. The model is in a folder named Finite element model. If reviewers and editors want to repeat the experiment or verify the reliability of experimental modal data, they can directly use the finite element model provided by the author. It is worth noting that COMSOL software is used in this study. The folder named Case 1 and Case 2 represent the results of the model under Case 1 and Case 2 boundary conditions, respectively. [file 3935803.f1.zip › 3935803.f1/BC2/FV/k5-__xz .png]

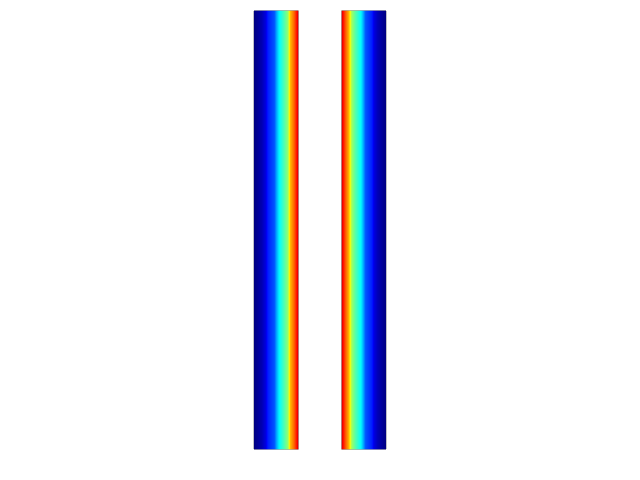

Supplement: Supplementary Materials — All the data and images were calculated by the finite element model. The model is in a folder named Finite element model. If reviewers and editors want to repeat the experiment or verify the reliability of experimental modal data, they can directly use the finite element model provided by the author. It is worth noting that COMSOL software is used in this study. The folder named Case 1 and Case 2 represent the results of the model under Case 1 and Case 2 boundary conditions, respectively. [file 3935803.f1.zip › 3935803.f1/BC2/FV/k5-__yz .png]

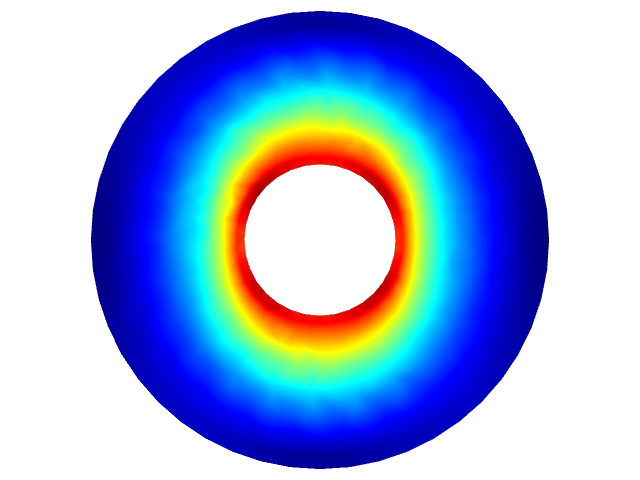

Supplement: Supplementary Materials — All the data and images were calculated by the finite element model. The model is in a folder named Finite element model. If reviewers and editors want to repeat the experiment or verify the reliability of experimental modal data, they can directly use the finite element model provided by the author. It is worth noting that COMSOL software is used in this study. The folder named Case 1 and Case 2 represent the results of the model under Case 1 and Case 2 boundary conditions, respectively. [file 3935803.f1.zip › 3935803.f1/BC2/FV/k6-__ .png]

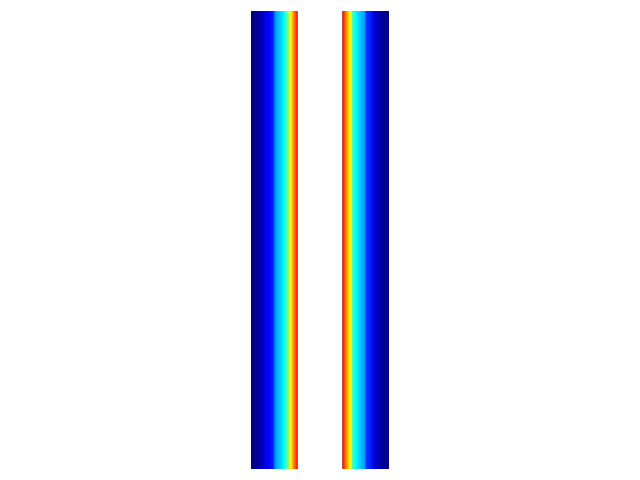

Supplement: Supplementary Materials — All the data and images were calculated by the finite element model. The model is in a folder named Finite element model. If reviewers and editors want to repeat the experiment or verify the reliability of experimental modal data, they can directly use the finite element model provided by the author. It is worth noting that COMSOL software is used in this study. The folder named Case 1 and Case 2 represent the results of the model under Case 1 and Case 2 boundary conditions, respectively. [file 3935803.f1.zip › 3935803.f1/BC2/FV/k6-__xz .png]

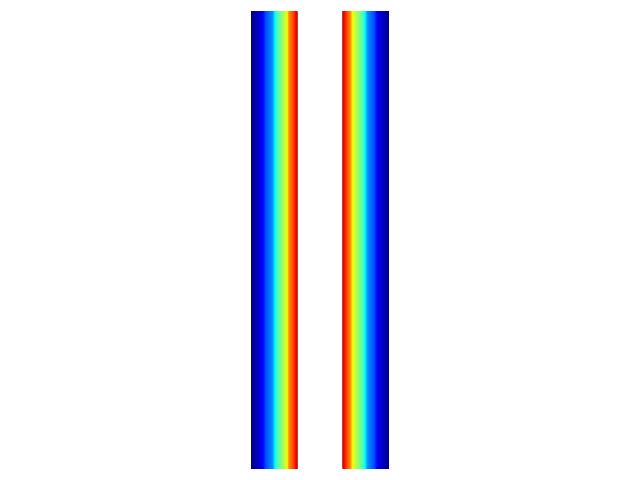

Supplement: Supplementary Materials — All the data and images were calculated by the finite element model. The model is in a folder named Finite element model. If reviewers and editors want to repeat the experiment or verify the reliability of experimental modal data, they can directly use the finite element model provided by the author. It is worth noting that COMSOL software is used in this study. The folder named Case 1 and Case 2 represent the results of the model under Case 1 and Case 2 boundary conditions, respectively. [file 3935803.f1.zip › 3935803.f1/BC2/FV/k6-__yz .png]

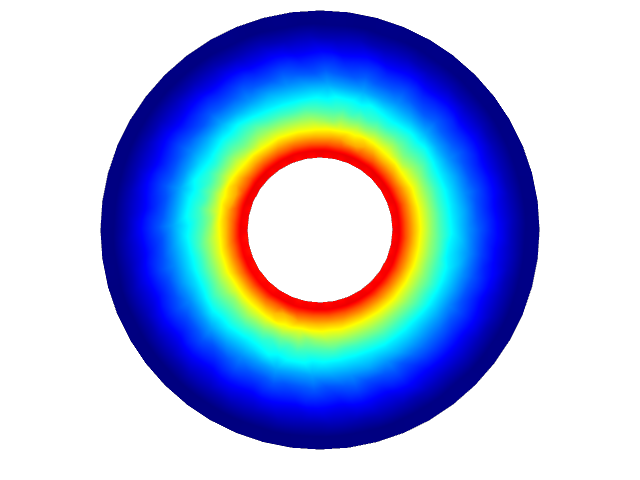

Supplement: Supplementary Materials — All the data and images were calculated by the finite element model. The model is in a folder named Finite element model. If reviewers and editors want to repeat the experiment or verify the reliability of experimental modal data, they can directly use the finite element model provided by the author. It is worth noting that COMSOL software is used in this study. The folder named Case 1 and Case 2 represent the results of the model under Case 1 and Case 2 boundary conditions, respectively. [file 3935803.f1.zip › 3935803.f1/BC2/FV/k7-__ .png]

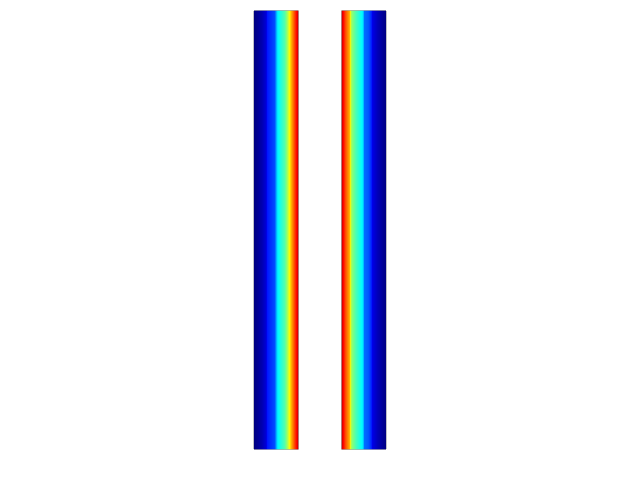

Supplement: Supplementary Materials — All the data and images were calculated by the finite element model. The model is in a folder named Finite element model. If reviewers and editors want to repeat the experiment or verify the reliability of experimental modal data, they can directly use the finite element model provided by the author. It is worth noting that COMSOL software is used in this study. The folder named Case 1 and Case 2 represent the results of the model under Case 1 and Case 2 boundary conditions, respectively. [file 3935803.f1.zip › 3935803.f1/BC2/FV/k7-__xz .png]

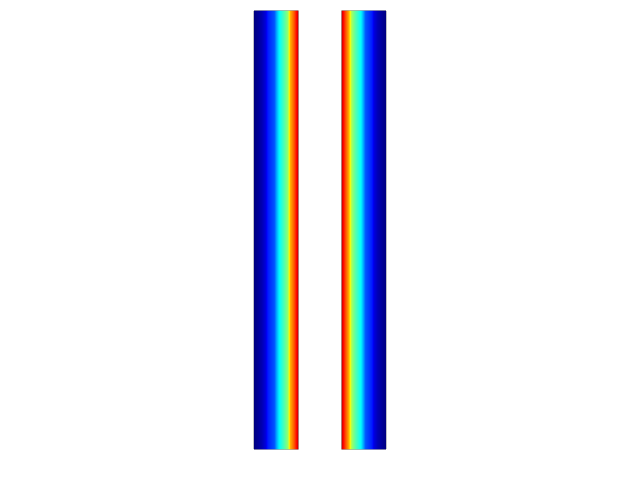

Supplement: Supplementary Materials — All the data and images were calculated by the finite element model. The model is in a folder named Finite element model. If reviewers and editors want to repeat the experiment or verify the reliability of experimental modal data, they can directly use the finite element model provided by the author. It is worth noting that COMSOL software is used in this study. The folder named Case 1 and Case 2 represent the results of the model under Case 1 and Case 2 boundary conditions, respectively. [file 3935803.f1.zip › 3935803.f1/BC2/FV/k7-__yz .png]

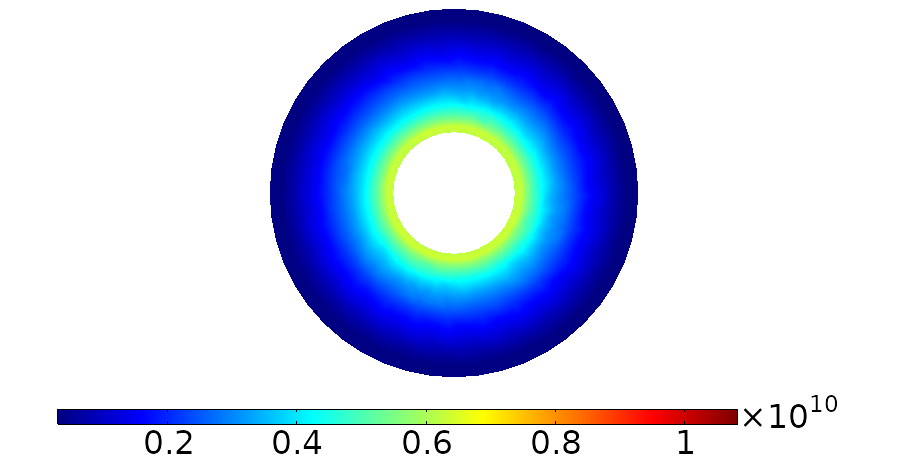

Supplement: Supplementary Materials — All the data and images were calculated by the finite element model. The model is in a folder named Finite element model. If reviewers and editors want to repeat the experiment or verify the reliability of experimental modal data, they can directly use the finite element model provided by the author. It is worth noting that COMSOL software is used in this study. The folder named Case 1 and Case 2 represent the results of the model under Case 1 and Case 2 boundary conditions, respectively. [file 3935803.f1.zip › 3935803.f1/BC2/PG/__.png]

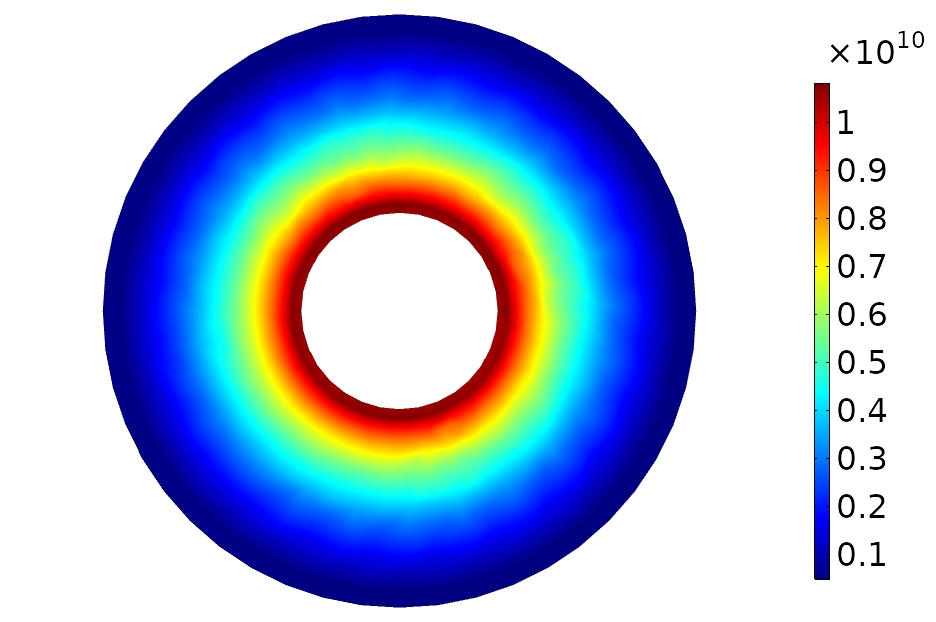

Supplement: Supplementary Materials — All the data and images were calculated by the finite element model. The model is in a folder named Finite element model. If reviewers and editors want to repeat the experiment or verify the reliability of experimental modal data, they can directly use the finite element model provided by the author. It is worth noting that COMSOL software is used in this study. The folder named Case 1 and Case 2 represent the results of the model under Case 1 and Case 2 boundary conditions, respectively. [file 3935803.f1.zip › 3935803.f1/BC2/PG/__1.png]

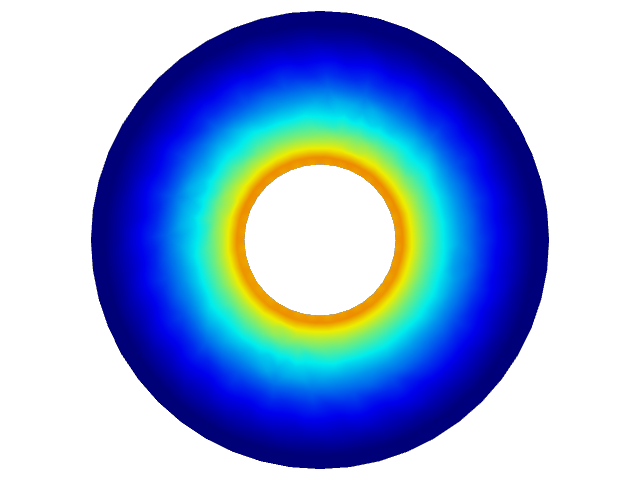

Supplement: Supplementary Materials — All the data and images were calculated by the finite element model. The model is in a folder named Finite element model. If reviewers and editors want to repeat the experiment or verify the reliability of experimental modal data, they can directly use the finite element model provided by the author. It is worth noting that COMSOL software is used in this study. The folder named Case 1 and Case 2 represent the results of the model under Case 1 and Case 2 boundary conditions, respectively. [file 3935803.f1.zip › 3935803.f1/BC2/PG/k1____.png]

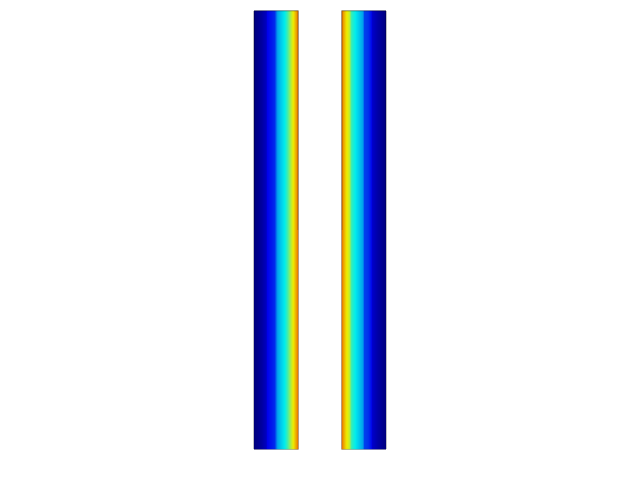

Supplement: Supplementary Materials — All the data and images were calculated by the finite element model. The model is in a folder named Finite element model. If reviewers and editors want to repeat the experiment or verify the reliability of experimental modal data, they can directly use the finite element model provided by the author. It is worth noting that COMSOL software is used in this study. The folder named Case 1 and Case 2 represent the results of the model under Case 1 and Case 2 boundary conditions, respectively. [file 3935803.f1.zip › 3935803.f1/BC2/PG/k1____xz.png]

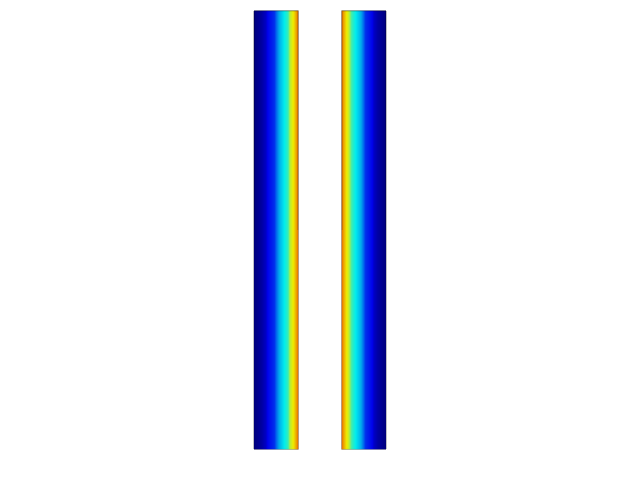

Supplement: Supplementary Materials — All the data and images were calculated by the finite element model. The model is in a folder named Finite element model. If reviewers and editors want to repeat the experiment or verify the reliability of experimental modal data, they can directly use the finite element model provided by the author. It is worth noting that COMSOL software is used in this study. The folder named Case 1 and Case 2 represent the results of the model under Case 1 and Case 2 boundary conditions, respectively. [file 3935803.f1.zip › 3935803.f1/BC2/PG/k1____yz.png]

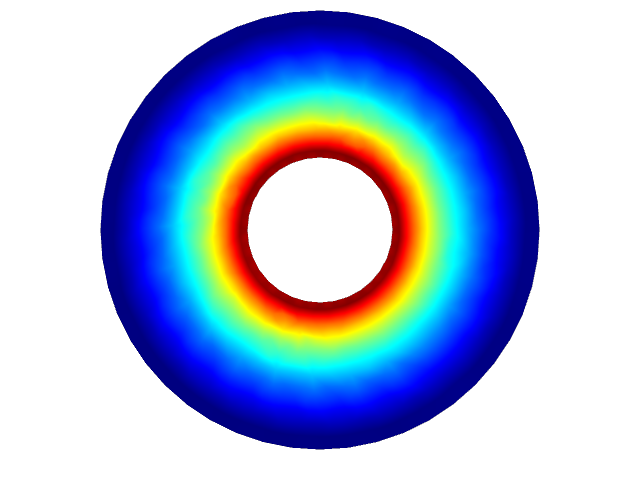

Supplement: Supplementary Materials — All the data and images were calculated by the finite element model. The model is in a folder named Finite element model. If reviewers and editors want to repeat the experiment or verify the reliability of experimental modal data, they can directly use the finite element model provided by the author. It is worth noting that COMSOL software is used in this study. The folder named Case 1 and Case 2 represent the results of the model under Case 1 and Case 2 boundary conditions, respectively. [file 3935803.f1.zip › 3935803.f1/BC2/PG/k2____.png]

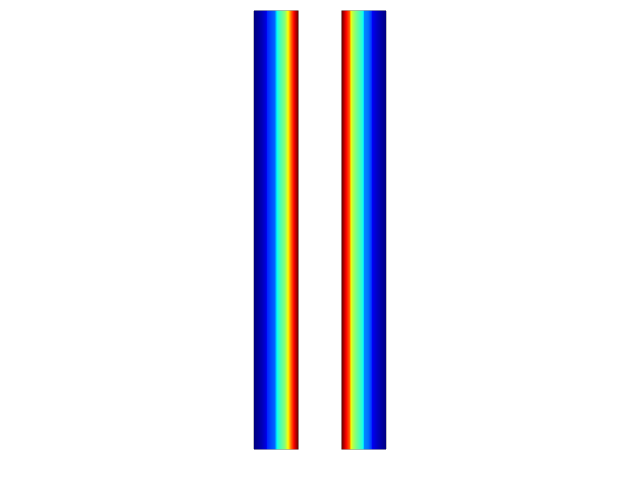

Supplement: Supplementary Materials — All the data and images were calculated by the finite element model. The model is in a folder named Finite element model. If reviewers and editors want to repeat the experiment or verify the reliability of experimental modal data, they can directly use the finite element model provided by the author. It is worth noting that COMSOL software is used in this study. The folder named Case 1 and Case 2 represent the results of the model under Case 1 and Case 2 boundary conditions, respectively. [file 3935803.f1.zip › 3935803.f1/BC2/PG/k2____xz.png]

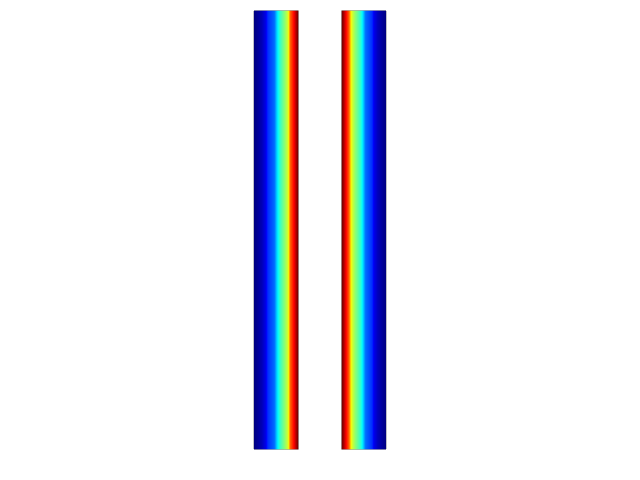

Supplement: Supplementary Materials — All the data and images were calculated by the finite element model. The model is in a folder named Finite element model. If reviewers and editors want to repeat the experiment or verify the reliability of experimental modal data, they can directly use the finite element model provided by the author. It is worth noting that COMSOL software is used in this study. The folder named Case 1 and Case 2 represent the results of the model under Case 1 and Case 2 boundary conditions, respectively. [file 3935803.f1.zip › 3935803.f1/BC2/PG/k2____yz.png]

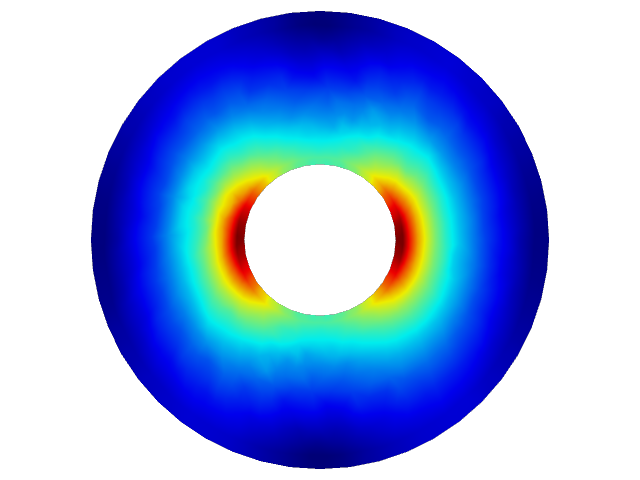

Supplement: Supplementary Materials — All the data and images were calculated by the finite element model. The model is in a folder named Finite element model. If reviewers and editors want to repeat the experiment or verify the reliability of experimental modal data, they can directly use the finite element model provided by the author. It is worth noting that COMSOL software is used in this study. The folder named Case 1 and Case 2 represent the results of the model under Case 1 and Case 2 boundary conditions, respectively. [file 3935803.f1.zip › 3935803.f1/BC2/PG/k3____.png]

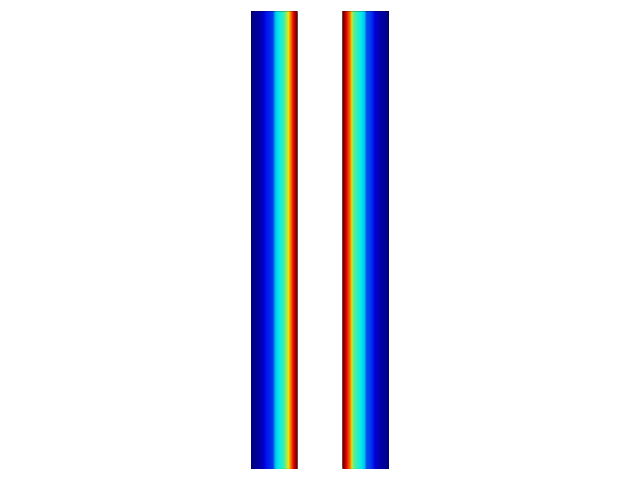

Supplement: Supplementary Materials — All the data and images were calculated by the finite element model. The model is in a folder named Finite element model. If reviewers and editors want to repeat the experiment or verify the reliability of experimental modal data, they can directly use the finite element model provided by the author. It is worth noting that COMSOL software is used in this study. The folder named Case 1 and Case 2 represent the results of the model under Case 1 and Case 2 boundary conditions, respectively. [file 3935803.f1.zip › 3935803.f1/BC2/PG/k3____xz.png]

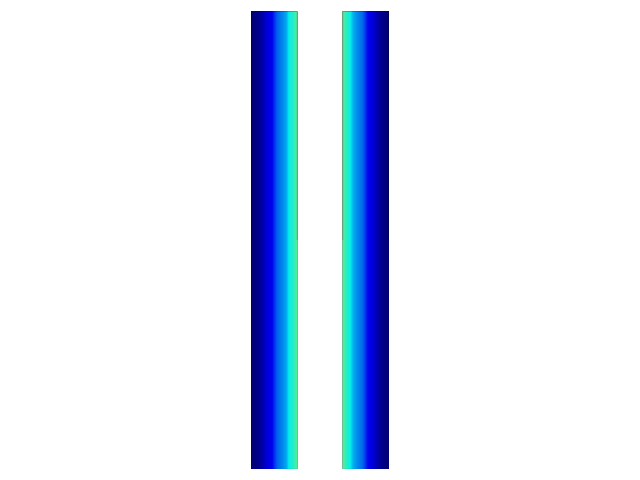

Supplement: Supplementary Materials — All the data and images were calculated by the finite element model. The model is in a folder named Finite element model. If reviewers and editors want to repeat the experiment or verify the reliability of experimental modal data, they can directly use the finite element model provided by the author. It is worth noting that COMSOL software is used in this study. The folder named Case 1 and Case 2 represent the results of the model under Case 1 and Case 2 boundary conditions, respectively. [file 3935803.f1.zip › 3935803.f1/BC2/PG/k3____yz.png]

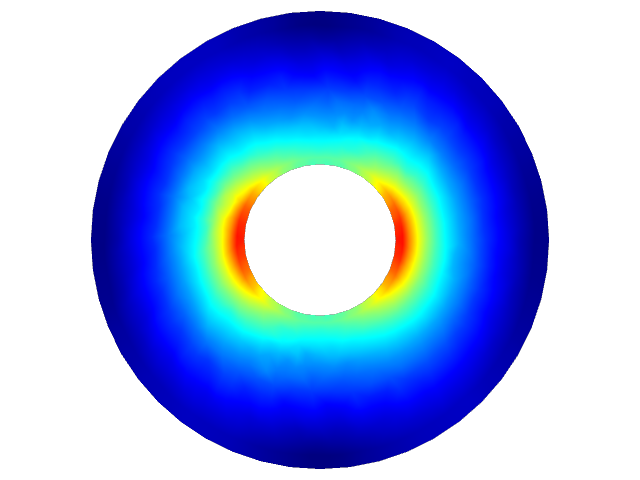

Supplement: Supplementary Materials — All the data and images were calculated by the finite element model. The model is in a folder named Finite element model. If reviewers and editors want to repeat the experiment or verify the reliability of experimental modal data, they can directly use the finite element model provided by the author. It is worth noting that COMSOL software is used in this study. The folder named Case 1 and Case 2 represent the results of the model under Case 1 and Case 2 boundary conditions, respectively. [file 3935803.f1.zip › 3935803.f1/BC2/PG/k4____.png]

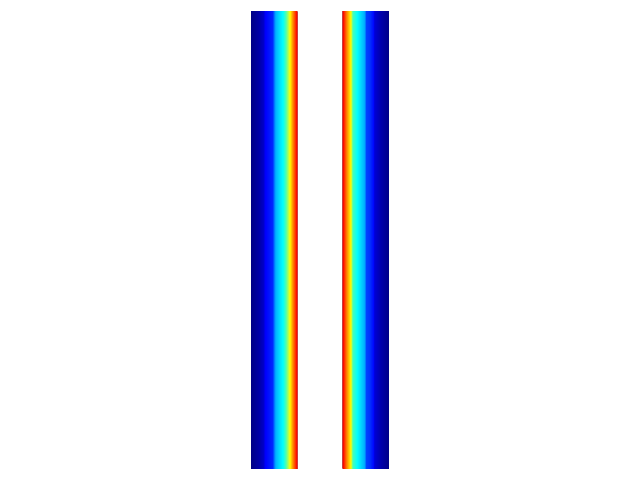

Supplement: Supplementary Materials — All the data and images were calculated by the finite element model. The model is in a folder named Finite element model. If reviewers and editors want to repeat the experiment or verify the reliability of experimental modal data, they can directly use the finite element model provided by the author. It is worth noting that COMSOL software is used in this study. The folder named Case 1 and Case 2 represent the results of the model under Case 1 and Case 2 boundary conditions, respectively. [file 3935803.f1.zip › 3935803.f1/BC2/PG/k4____xz.png]

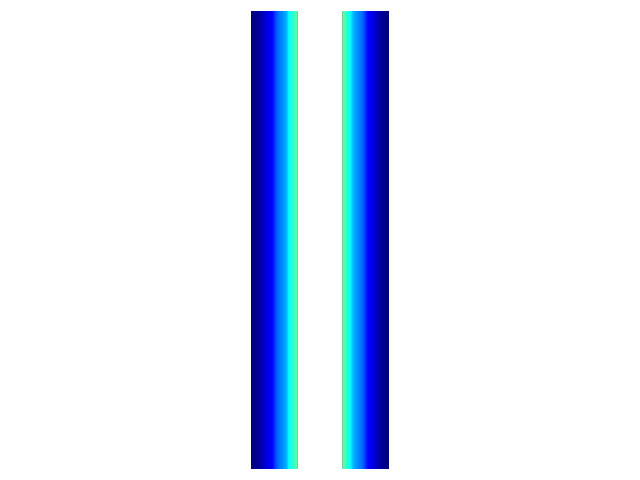

Supplement: Supplementary Materials — All the data and images were calculated by the finite element model. The model is in a folder named Finite element model. If reviewers and editors want to repeat the experiment or verify the reliability of experimental modal data, they can directly use the finite element model provided by the author. It is worth noting that COMSOL software is used in this study. The folder named Case 1 and Case 2 represent the results of the model under Case 1 and Case 2 boundary conditions, respectively. [file 3935803.f1.zip › 3935803.f1/BC2/PG/k4____yz.png]

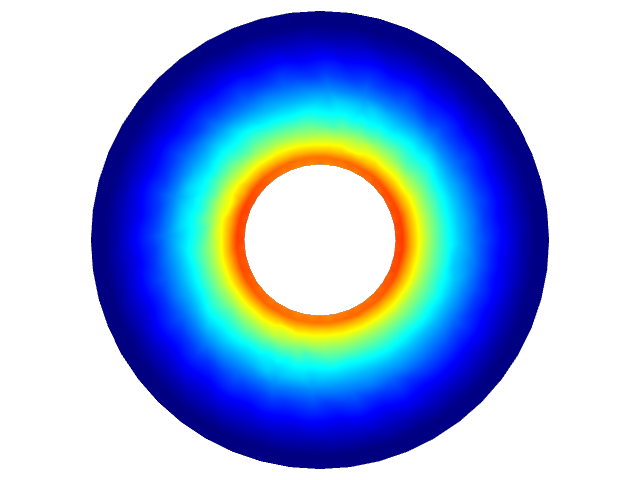

Supplement: Supplementary Materials — All the data and images were calculated by the finite element model. The model is in a folder named Finite element model. If reviewers and editors want to repeat the experiment or verify the reliability of experimental modal data, they can directly use the finite element model provided by the author. It is worth noting that COMSOL software is used in this study. The folder named Case 1 and Case 2 represent the results of the model under Case 1 and Case 2 boundary conditions, respectively. [file 3935803.f1.zip › 3935803.f1/BC2/PG/k5____.png]

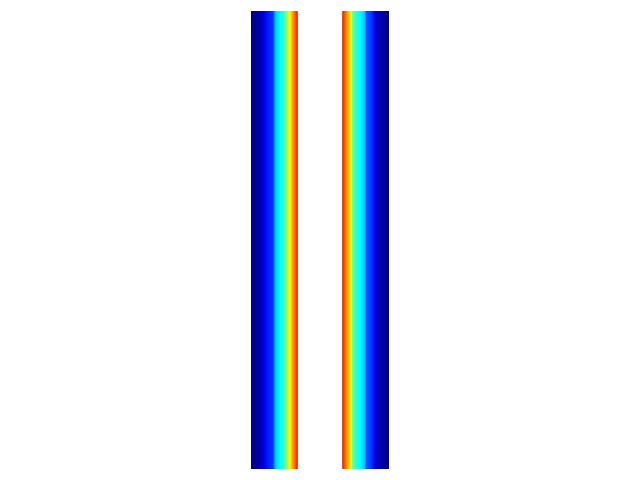

Supplement: Supplementary Materials — All the data and images were calculated by the finite element model. The model is in a folder named Finite element model. If reviewers and editors want to repeat the experiment or verify the reliability of experimental modal data, they can directly use the finite element model provided by the author. It is worth noting that COMSOL software is used in this study. The folder named Case 1 and Case 2 represent the results of the model under Case 1 and Case 2 boundary conditions, respectively. [file 3935803.f1.zip › 3935803.f1/BC2/PG/k5____xz.png]

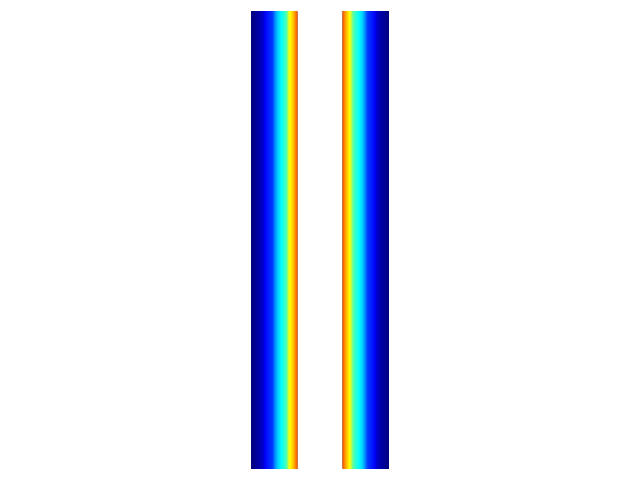

Supplement: Supplementary Materials — All the data and images were calculated by the finite element model. The model is in a folder named Finite element model. If reviewers and editors want to repeat the experiment or verify the reliability of experimental modal data, they can directly use the finite element model provided by the author. It is worth noting that COMSOL software is used in this study. The folder named Case 1 and Case 2 represent the results of the model under Case 1 and Case 2 boundary conditions, respectively. [file 3935803.f1.zip › 3935803.f1/BC2/PG/k5____yz.png]

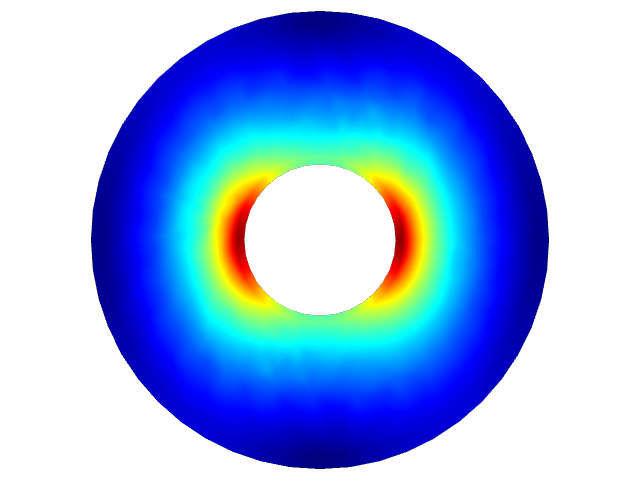

Supplement: Supplementary Materials — All the data and images were calculated by the finite element model. The model is in a folder named Finite element model. If reviewers and editors want to repeat the experiment or verify the reliability of experimental modal data, they can directly use the finite element model provided by the author. It is worth noting that COMSOL software is used in this study. The folder named Case 1 and Case 2 represent the results of the model under Case 1 and Case 2 boundary conditions, respectively. [file 3935803.f1.zip › 3935803.f1/BC2/PG/k6____.png]

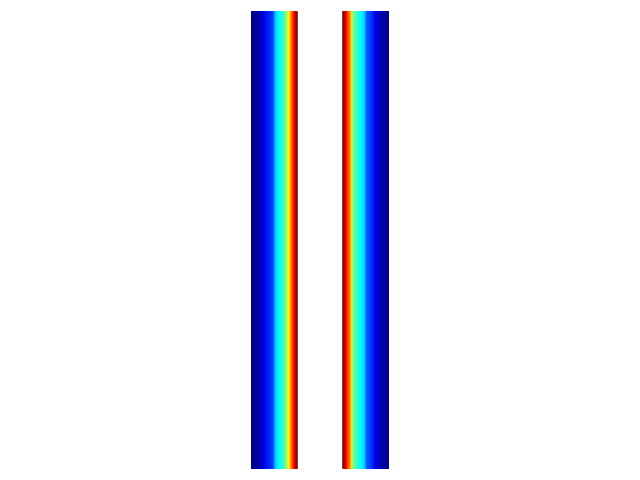

Supplement: Supplementary Materials — All the data and images were calculated by the finite element model. The model is in a folder named Finite element model. If reviewers and editors want to repeat the experiment or verify the reliability of experimental modal data, they can directly use the finite element model provided by the author. It is worth noting that COMSOL software is used in this study. The folder named Case 1 and Case 2 represent the results of the model under Case 1 and Case 2 boundary conditions, respectively. [file 3935803.f1.zip › 3935803.f1/BC2/PG/k6____xz.png]

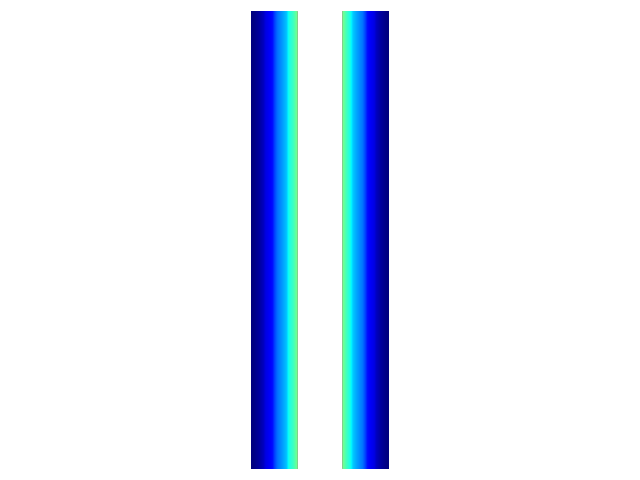

Supplement: Supplementary Materials — All the data and images were calculated by the finite element model. The model is in a folder named Finite element model. If reviewers and editors want to repeat the experiment or verify the reliability of experimental modal data, they can directly use the finite element model provided by the author. It is worth noting that COMSOL software is used in this study. The folder named Case 1 and Case 2 represent the results of the model under Case 1 and Case 2 boundary conditions, respectively. [file 3935803.f1.zip › 3935803.f1/BC2/PG/k6____yz.png]

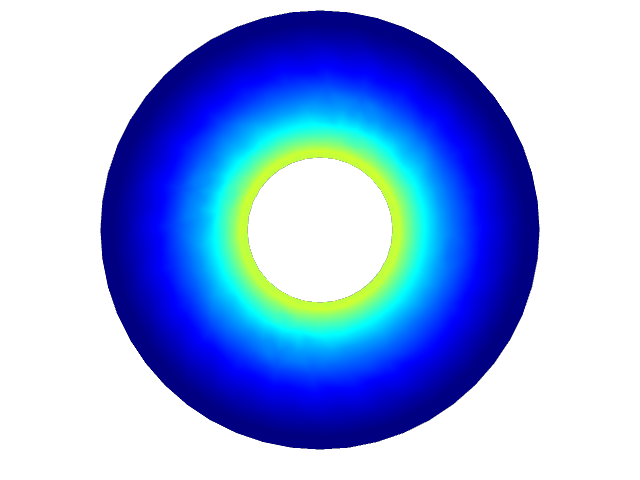

Supplement: Supplementary Materials — All the data and images were calculated by the finite element model. The model is in a folder named Finite element model. If reviewers and editors want to repeat the experiment or verify the reliability of experimental modal data, they can directly use the finite element model provided by the author. It is worth noting that COMSOL software is used in this study. The folder named Case 1 and Case 2 represent the results of the model under Case 1 and Case 2 boundary conditions, respectively. [file 3935803.f1.zip › 3935803.f1/BC2/PG/k7____.png]

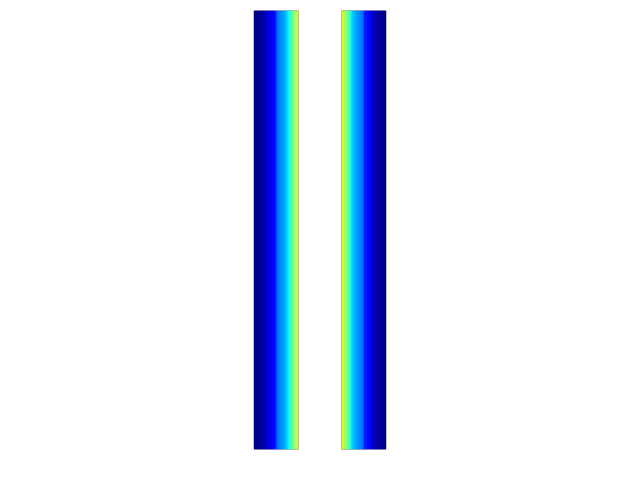

Supplement: Supplementary Materials — All the data and images were calculated by the finite element model. The model is in a folder named Finite element model. If reviewers and editors want to repeat the experiment or verify the reliability of experimental modal data, they can directly use the finite element model provided by the author. It is worth noting that COMSOL software is used in this study. The folder named Case 1 and Case 2 represent the results of the model under Case 1 and Case 2 boundary conditions, respectively. [file 3935803.f1.zip › 3935803.f1/BC2/PG/k7____xz.png]
